# Spatiotemporal Dynamics of the Molecular Expression Pattern and Intercellular Interactions in the Glial Scar Response to Spinal Cord Injury

**DOI:** 10.1007/s12264-022-00897-8

**Published:** 2022-07-05

**Authors:** Leilei Gong, Yun Gu, Xiaoxiao Han, Chengcheng Luan, Chang Liu, Xinghui Wang, Yufeng Sun, Mengru Zheng, Mengya Fang, Shuhai Yang, Lai Xu, Hualin Sun, Bin Yu, Xiaosong Gu, Songlin Zhou

**Affiliations:** Key Laboratory of Neuroregeneration of Jiangsu and Ministry of Education, NMPA Key Laboratory for Research and Evaluation of Tissue Engineering Technology Products, Jiangsu Clinical Medicine Center of Tissue Engineering and Nerve Injury Repair, Co-Innovation Center of Neuroregeneration, Nantong University, Nantong, 226001 China

**Keywords:** Spinal cord injury, Glial scar, Spatial transcriptomics, Microenvironment, Therapeutic strategy, Salvianolic acid

## Abstract

Nerve regeneration in adult mammalian spinal cord is poor because of the lack of intrinsic regeneration of neurons and extrinsic factors – the glial scar is triggered by injury and inhibits or promotes regeneration. Recent technological advances in spatial transcriptomics (ST) provide a unique opportunity to decipher most genes systematically throughout scar formation, which remains poorly understood. Here, we first constructed the tissue-wide gene expression patterns of mouse spinal cords over the course of scar formation using ST after spinal cord injury from 32 samples. Locally, we profiled gene expression gradients from the leading edge to the core of the scar areas to further understand the scar microenvironment, such as neurotransmitter disorders, activation of the pro-inflammatory response, neurotoxic saturated lipids, angiogenesis, obstructed axon extension, and extracellular structure re-organization. In addition, we described 21 cell transcriptional states during scar formation and delineated the origins, functional diversity, and possible trajectories of subpopulations of fibroblasts, glia, and immune cells. Specifically, we found some regulators in special cell types, such as Thbs1 and Col1a2 in macrophages, CD36 and Postn in fibroblasts, Plxnb2 and Nxpe3 in microglia, Clu in astrocytes, and CD74 in oligodendrocytes. Furthermore, salvianolic acid B, a blood–brain barrier permeation and CD36 inhibitor, was administered after surgery and found to remedy fibrosis. Subsequently, we described the extent of the scar boundary and profiled the bidirectional ligand-receptor interactions at the neighboring cluster boundary, contributing to maintain scar architecture during gliosis and fibrosis, and found that GPR37L1_PSAP, and GPR37_PSAP were the most significant gene-pairs among microglia, fibroblasts, and astrocytes. Last, we quantified the fraction of scar-resident cells and proposed four possible phases of scar formation: macrophage infiltration, proliferation and differentiation of scar-resident cells, scar emergence, and scar stationary. Together, these profiles delineated the spatial heterogeneity of the scar, confirmed the previous concepts about scar architecture, provided some new clues for scar formation, and served as a valuable resource for the treatment of central nervous system injury.

**Supplementary Information:**

The online version contains supplementary material available at 10.1007/s12264-022-00897-8.

## Introduction

Spinal cord injury (SCI) is mostly caused by accidents and is a lifelong, traumatic injury with a tremendous social and economic impact [1–4]. More worryingly, clinical therapeutic methods to promote spinal cord regeneration are too limited and the outcomes are complicated, closely depending on the level of the injury [5–7]. The pathophysiological processes of SCI involve a primary injury and the formation of a glial scar [8]. Although it is widely accepted that the glial scar inhibits axon regeneration and protects spared neural tissues from injury, the exact role of the glial scar in SCI remains poorly understood [9]. During ~2 weeks, the glial scar becomes mature, and this is associated with many cellular and extracellular components and their complex interactions [10]. The cellular components of the glial scar mainly consist of macrophages, fibroblasts, microglia, astrocytes, oligodendrocytes, NG2^+^ oligodendrocyte precursor cells (OPCs), and ependymal cells. The extracellular matrix (ECM) includes chondroitin sulfate proteoglycans, myelin-associated glycoprotein, fibronectin, oligodendrocyte myelin glycoprotein, matrix glycoprotein tenascin C, and hyaluronan fragments [11–13]. To date, research on the cellular mechanisms and molecular regulation of these components are still in progress [14]. Interestingly, the spiny mouse (Acomys cahirinus) has emerged as a curious exception as it sustains scarless regeneration and functional recovery after complete spinal cord transection [15]. Therefore, it is essential to comprehensively understand the above events in order to develop more effective treatments for SCI [16].

Rapid advances in single-cell RNA sequencing (scRNA-seq) technologies have permitted the use of single-cell transcriptional profiling to investigate the cellular heterogeneity in the central nervous system (CNS), advancing our knowledge of CNS disease. For example, astrocytes, a major cell type found throughout the CNS, have a wide array of functions in the modulation of synaptic transmission, regulation of blood flow, formation of the blood-brain barrier, and the metabolic support of other brain resident cells, yet the character of their cellular heterogeneity and how it manages these diverse roles remains unclear. Using a fluorescence-activated cell sorting-based strategy, five distinct astrocyte subpopulations have been identified across three brain regions that show extensive molecular diversity [17]. In addition, five distinct astrocyte subtypes have been identified in adult mouse cortex and hippocampus [18]. Recently, a population of disease-associated astrocytes was identified in a mouse model of Alzheimer's disease using scRNA-seq. These disease-associated astrocytes appear at early stages in the disease and increase in abundance with disease progression [19]. Intriguingly, the heterogeneity and interactions of intra-glial scar gene expression have been documented using scRNA-Seq at the level of individual cells [20, 21].

A common feature of transcriptome-wide scRNA-seq studies is that they do not address the spatial patterns of gene expression. Although computational inference can partially circumvent the lack of spatial information in scRNA-Seq data [22, 23], we still need alternative methods that provide positional information on the different cell types involved in the process; this is necessary for a comprehensive understanding of glial scar dynamics [12, 24]. Although current *in situ* sequencing techniques, such as expansion-assisted iterative fluorescence *in situ* hybridization, have been exploited [25–29], these approaches have limitations. They require prior knowledge about the genes of interest, and use padlock probes and rolling circle amplification in tissue sections to target known genes. The biggest flaw is that this approach can only detect small numbers of marker genes [25, 30]. The spatial dimensions of entire transcriptomes remain unexplored in the glial scar, along with the spinal cord micro-environment [20, 21].

Spatial transcriptomics (ST) is an untargeted technology that produces quantitative transcriptome-wide RNA sequencing data and determines the quantitative spatial distribution of polyadenylated transcripts in a tissue section using barcoded oligo-dT DNA capture probe arrays corresponding to histological imaging [31]. The throughput of the approach is substantially greater than that of other spatially-resolved methods. It produces spatial maps based on RNA-seq data and has broad applicability [32–34]. For example, studies on the brain [31, 35, 36], of spinal cord neurodegeneration [37], and the development of the human heart [38, 39] have offered spatial cell-specific findings that could not have been accomplished using traditional tissue homogenates. It contributes to confirming regional markers and cell-type identification based on scRNA-seq and ST.

**Fig. 1:**
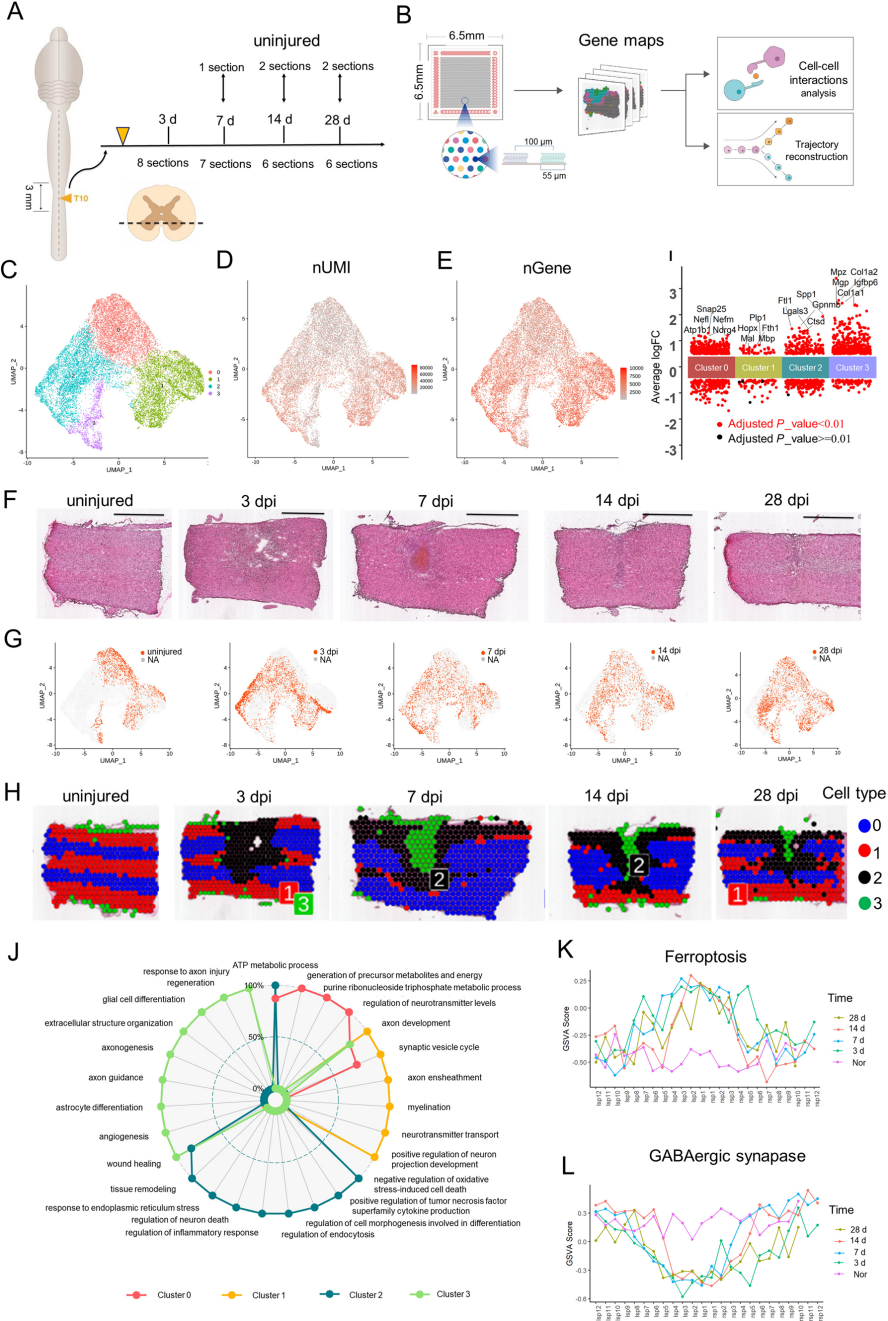
Generation of a spatiotemporal transcriptional atlas of the mouse glial scar. **A** Overview of the study design for glia scar formation and location of sections used in this study. **B** Experimental workflow and analysis for spatial RNA-seq of the glia scar in adult mouse at four stages of scar maturation after T10 right lateral hemisection. The spatial microarrays had 4,992 spatially-barcoded spots 55 µm in diameter and a 100-µm center-to-center distance. The Spatial Transcriptomic (ST) procedure yielded matrices with read counts for every gene in every spot, which were then decomposed by a set of factors (“cell types”). **C** Uniform Manifold Approximation and Projection (UMAP) plot of spots from all sections visualized using Seurat package and profiling the cell clusters. (**D**, **E**) UMAP profiling the number of expressed unique molecular identifier (nUMI) and genes (nGene). **F** H&E staining images showing the changes in pathological morphology of glia scar maturation (scale bar, 1,000 µm). **G** UMAP profiles of the spots at different times after injury. **H** UMAP spots mapped to their spatial locations. **I** The top 5 highest differentially-expressed genes in each cluster. **J** Gene Ontology analysis data enriched for each cluster. **K** GSVA scores of the ferroptosis signaling pathway counted at three stages in Layer 3. **L** GSVA scores of the GABAergic synapse pathway counted at three stages in Layer 3.

Here, we applied ST to spatially profile gene expression in thoracic (T10) spinal cord tissue sections from WT mice to present a spatiotemporal atlas that systematically describes the spatial archetypes and cellular heterogeneity of scar formation [10, 20] (Fig. 1A). We obtained the molecular signatures of multiple cell transcriptional states and pseudotime trajectory at three stages: the early acute [3 days post-injury (dpi)], subacute (7 dpi), and intermediate stages (14 and 28 dpi) [10, 20]. By assessing the expression of bidirectional ligand-receptor pairs and the spatial distribution of different subpopulations, we described architecture and micro-environment of the scar. Furthermore, salvianolic acid B (SAB) was found to remedy fibrosis based on CD36 ST maps in the fibrosis scar. As the first ST analysis of the spinal cord scar, our transcriptomic dataset confirmed previous concepts, added to the general knowledge, contributed to the comprehensive decoding of the glial scar, and explored the potential therapeutic strategies for clinical application.

## Materials and Methods

### Mice and Surgery

Sixty female mice (8 weeks old, C57BL/6J) were randomly divided into five groups (*n =* 12). The mice were anaesthetized by isoflurane. The procedure for T10 right lateral hemisection was similar to that described previously [40]. Briefly, a midline incision was made over the thoracic vertebrae and a T10 laminectomy was performed. To produce a right lateral hemisection injury, the tip of an optimum knife (BVI Beaver, Oakville, Canada) was carefully inserted into the posterior median sulcus of the spinal cord and then gently scraped across the bone on the ventral side so as to not spare any tissue ventrally or laterally. The muscle layers were sutured, and then the skin was secured with wound clips. The mice were placed on soft bedding on a warming blanket held at 37°C until fully awake and given ibuprofen for pain relief. All the animal experimental were conducted in accordance within situational animal care guidelines and given ethical approval by the Administration Committee of Experimental Animals, Jiangsu Province, China (20150304-004).

### Spatial Transcriptomic Tissue Imaging, Library Preparation, and Sequencing

Libraries were prepared using Spatial Transcriptomic Library Preparation Glass Slides following the manufacturer’s instructions. In brief, frozen samples were embedded in OCT (TissueTek, Sakura) and stored at −80°C until cryosection. For cryo-sectioning, samples were equilibrated to –22 °C and cut at 10 μm on a Leica CM3050 S cryostat. The sections were placed on chilled Visium Tissue Optimization Slides (3000394, 10× Genomics) and Visium Spatial Gene Expression Slides (2000233, 10× Genomics), and adhered by warming the back of the slide. The sections were then fixed in chilled methanol and stained with hematoxylin and eosin Y according to the Visium Spatial Gene Expression User Guide (CG000239 Rev D, 10× Genomics). For sequencing, the tissue was permeabilized for 24 min, which was selected as the optimal time based on tissue optimization time-course experiments. Libraries were prepared according to the Visium Spatial Gene Expression User Guide. cDNA was synthesized overnight using SuperScript III Reverse Transcriptase in the presence of actinomycin. Libraries were loaded at 300 pM and sequenced on a NovaSeq 6000 System (Illumina) using a NovaSeq S4 Reagent Kit (Illumina). Sequencing was performed using the following read protocol: read 1, 28 cycles; i7 index, 10 cycles; i5 index, 10 cycles; read 2, 90 cycles. The sequencing data are available with NCBI accession number GSE195783.

To study the effects of injury on motor neurons, we selected a ventral horizontal section in each mouse that crossed the anterior horn and had a better scar shape. After taking these factors into account, we finally selected 8 sections for 3 dpi, 7 for 7 dpi, and 6 for 14 and 28 dpi. We used 10× Visium in two batches to assess these samples.

### Spatial Transcriptomics Analysis

Reads were mapped against the Mouse Genome (mm10) and gene-spot matrixes were generated by Space Ranger-1.2.0 (10× Genomics). The number of genes with at least 163 unique molecular identifiers (UMIs) was counted in each tissue -covered spot. The combined data were normalized with Seurat sctransform. We computed principal components (PCs) to perform dimensionality reduction processing on the combined data, and selected the top 30 PCs for downstream data analysis. We performed graph-based clustering across all samples and displayed them by UMAP. Marker genes of the five clusters were analyzed by Seurat 3.2.2. The functional enrichment marker gene and Gene ontology (GO) and Kyoto Encyclopedia of Genes and Genomes (KEGG) pathway analysis in each cluster were performed using the cluster Profiler package in R.

### Gene Set Variation Analysis (GSVA)

The pathway activities of selected spots were quantified by applying the GSVA package [41]. In order to show the expression of different genes in space, we integrated the physical coordinates of 4992 points on the spatial transcriptome chip with the coordinate information of the corresponding spot points, and then used the R language ggplot2 package to project them into two-dimensional space. The UMI value in each spot represented the expression abundance expression (Ei) of each gene. Here, i indicates the expression vector of each spot:

$$ {\text{Ei}} = {\text{log}}[{\text{UMI}}({\text{i}}) + { 1}] $$

The degree of activation of the signal pathway is reflected in the score value.

### Pseudotime Trajectory

Pseudotime trajectories for selected subclusters were constructed using the R package Monocle v2.14. 0 [42]. The raw counts for cells were extracted and normalized by the estimate size factors and estimate dispersion functions with default parameters. Genes detected in >50 spots and with an average expression >0.1 were retained. Differential expression analysis was carried out using the differential GeneTest function with a model against the pseudotime. Variable genes with the lowest adjusted q value <0.001 were clustered and plotted in the heatmap. Cell functions and the trajectory were constructed by the reduce dimension function with default parameters.

### Co-expression Network Analysis

The first co-expression networks were constructed using weighted correlation network analysis the (WGCNA) (v1.66) package in R [43]. After filtering genes, their expression values were imported into WGCNA to construct co-expression modules using the automatic network construction function. TOM Type was unsigned and minModule Size was 8. Genes were clustered into 14 correlated modules. To identify biologically significant modules, module eigengenes were used to calculate the correlation coefficient with samples or sample traits. Intramodular connectivity and the degree of module correlation of each gene were calculated using the R package of WGCNA, and genes with high connectivity tended to be hub genes that might have important functions. Correlation analysis was performed using the eigengene module with data for specific traits or phenotypes. Pearson correlations between each gene and trait data under the module were also calculated for the most relevant module (positive and negative correlations) corresponding to each data phenotype, and a gene significance value (GS) was obtained. Other unbiased co-expression networks were constructed using the method described previously [37]. The first 2000 highly-variable variables in all spots were normalized, and the correlations were calculated as the Pearson correlation coefficient. The co-expression modules were obtained by k-mean clustering.

### Ligand-Receptor Interaction Analysis

To infer potential cellular crosstalk between different cell types, we adapted the method used in CellPhoneDB for single-cell transcriptome data [44]. Enriched receptor–ligand interactions were predicted between two cell types based on the expression of a receptor by one cell type and a ligand by another cell type. Only receptors and ligands expressed more than a specified threshold among the cells in a specific cluster were considered significant (default is 0.1). The pairwise comparisons between all neighbor cell types in the scar were then calculated. First, we determined the mean of the average receptor expression level in a cluster and the average ligand expression level in the interacting neighbor cluster by randomly permuting the cluster labels of all cells. The *P* value for the likelihood of cell-type specificity of a given receptor–ligand complex was obtained by calculating the proportion of the means which were equal to or higher than the actual mean.

### SAB Treatment and Functional Evaluation

Thirty female mice (8 weeks old, C57BL/6J) were randomly divided into five groups (*n =* 6). To remedy fibrosis, injured mice were administered 100 μL intraperitoneal injections of either salvianolic acid B (Sigma) at 50, 100, or 200 µg/mL in PBS, or PBS alone, every day until 8 weeks after surgery. The SAB doses chosen were based on the literature [45]. Care was taken to avoid direct exposure of SAB to light. All functional analyses were performed at 2 days and weekly for 4 weeks post-infection. Hindlimb locomotor function was assessed using the Basso Mouse Scale (BMS) [46]. Briefly, patterns of hind limb movement, plantar stepping, and paw positions were observed in each animal. All mice were habituated to the BMS open-field arena in 10-min sessions every day for 1 week [15]. The locomotor score was assessed by two blinded independent observers.

### Immunostaining

Spinal cord sections were first fixed in 4% paraformaldehyde and then incubated overnight at 4ºC in the following primary antibodies: Iba1 (Wako, rabbit 1:100), GFAP (Abcam, chicken 1:100), P4HB (Abcam, mouse 1:100), F4/80 (Abcam, rabbit 1:200), CD68 (Novus Biological, mouse 1:100), laminin (Abcam, mouse 1:300), Fn1 (LSBio, sheep 1:100), collagen IV (SouthernBiotech, goat 1:100), Thbs1 (Invitrogen, mouse 1:100), Col1a2 (GeneTex, rabbit 1:100), and Nxpe3 (Abbexa, rabbit 1:100) diluted in PBS/0.3% Triton/5% normal donkey serum. After washing the sections with PBS, secondary Alexa Fluor-conjugated antibodies (Invitrogen) diluted in PBS/0.3% Triton were incubated for 2 h at room temperature. The slides were mounted with mounting medium containing DAPI (Beyotime Institute of Biotechnology, Shanghai, China) and kept at 4ºC until further microscopic analysis. The sections were visualized under a TCS SP2 confocal microscope (Leica-Microsystems GmbH, Mannheim, Germany) or a Zeiss M2 fluorescence microscope (Carl Zeiss Jena GmbH, Jena, Germany) and images from sections were processed in ImageJ.

### Spinal Cord Dehydration and Transparency

Dehydration, immunostaining, and clearing of the spinal cord were based on previous studies [47]. After washing off excess secondary antibodies, the tissue was gradually dehydrated in resistant glassware with incremental concentrations of tetrahydrofuran (TFH; 50% overnight, 80% for 1 h, and 100% for 2 h, v/v % in distilled water; Sigma-Aldrich). The tissue was incubated on a shaker at room temperature. Then, the remaining lipids were extracted with dichloromethane for 1 h and dibenzyl ether (DBE) was used for refractive index matching. Samples were kept in a full vial of DBE and protected from light during the whole process to reduce photo-bleaching of the fluorescence. Images were processed with a Lightsheet Microscope 18 [Nuohai Life Science (Shanghai) Co., Ltd].

## Result

### Overview of the Spatial Gene Expression in T10 Spinal Cord Sections

To ensure the uniformity of the wound area, we adapted the lateral hemisection model, rather than contusion [48]. A previous study revealed that the region of glial scar extended ~0.5 mm from the site of injury after SCI [49]. To decipher the cell types and regions of the scar as much as possible, we harvested spinal cord tissue 1.5 mm from the site of right lateral hemisection at T10 (Fig. 1A). Visium Spatial Gene Expression Slides were used to capture and profile the mRNAs in the spots of spinal cord sections at 3, 7, 14, and 28 days after the hemisection, which intended to cover the dynamic process of glial scar formation after injury [50]. Because each section contained up to 4,992 spots in its capture area and the size of the Visium Spatial Gene Expression Slides was 6.5 mm×6.5 mm (Fig. 1B), ~4 spinal cord samples could be placed on the same chip to capture more spots as shown in Fig. S1A. Supplementary Table S1 contains a summary of the data evaluation. Overall, 15,449 spots in the 32 sections were analyzed (Table S2, Fig. S1A and B). The data showed that the median sequencing depth of a single spot was ~219,442 (UMIs) and 4,875 genes (Table S2, Fig. S1C). The H&E staining images served as a reference for subsequent unsupervised analysis. We focused on the extent of the scar focus and boundary that contained the scar microenvironment, the identification of cell transcriptional states, the reconstruction of cell trajectories, and cell-cell interactions within the scar formation (Fig. 1B).

### Defining the Spatiotemporal Boundary of the Scar in the Injury Site

Integrated clustering analysis of ST from 32 samples using Seurat revealed 4 distinct clusters when visualized on a UMAP plot (Fig. 1C). These 4 clusters covered all cell types in the SCI site and adjacent uninjured spinal cord. The numbers of UMIs and genes in clusters 1, 2, and 3 were larger than that in cluster 0 (Fig. 1D, E). To better understand the spatial distribution of these cell types, we compared the H&E staining images (Fig. 1F) with their ST data (Fig. 1G) and mapped the clusters back to their spatial locations (Fig. 1H). These identified spatial patterns matched the changes in pathological morphology. Specifically, clusters 2 and 3 were characteristically distributed in the scar regions shown in H&E staining images, consistent with the larger UMI numbers. Based on these findings, we inferred that clusters 2 and 3 represented multiple cellular components that are known to comprise the glial scar [10, 20]. On the contrary, clusters 0 and 1 represented cell types that were similar to cells appearing in the uninjured spinal cord (Fig. 1E). Together, we described the approximate scope of the glial scar at three stages [10, 20] based on ST and in the subsequent analysis we only show cell spots involved in the scar.

Meanwhile, we listed the top 5 highest differentially-expressed genes (DEGs) in each cluster during this process from ST (Figs 1I, S1D). The highest DEGs provided a unique molecular signature for each cluster. For example, cluster 0 cells strongly expressed typical neuronal marker Snap25 and were likely to represent the neurons in the gray matter area of the uninjured spinal cord. Cluster 1 cells strongly expressed myelin-associated proteins [such as proteolipid protein 1 (Plp1), myelin basic protein (Mbp), and maltose binding protein (Mal)] and were likely to represent the oligodendrocytes in the white matter area of the uninjured spinal cord.

The highest DEGs in cluster 2 were secreted phosphoprotein-1 (Spp1), glycoprotein nonmetastatic melanoma protein B (Gpnmb), galactose-specific lectin 3 (Lgals3), and cathepsin D (Ctsd). Spp1, also known as osteopontin, is a broadly-expressed pleiotropic protein [51]. It has multiple functions in the pathophysiology of several inflammatory, degenerative, autoimmune, and oncologic diseases [52–54]. Spp1 plays both pro- and anti-inflammatory roles and contributes to tissue damage (the “Yin”) not only by recruiting harmful inflammatory cells to the lesion site, but also increasing their survival (the “Yang”) [52, 55]. For example, Spp1 is capable of stimulating mTOR activity [56] and promotes RGC regeneration [53]. In addition, Spp1 has been reported to stimulate the outgrowth of injured motor axons but not sensory neurons [57]. Our data showed the spatiotemporal distribution of Spp1 (Fig. S1E), which indeed appeared on some motor neurons and was significantly increased in the injury site, consistent with previous studies [52, 54, 55]. Lgals3 plays roles in numerous cellular functions including cell growth, cell adhesion, apoptosis, pre‑mRNA splicing, differentiation, transformation, angiogenesis, inflammation, T-cell regulation, host defense, and fibrosis [58, 59]. Based on the strong expression of the stress response and inflammatory regulators, cluster 2 cells were considered to be the glial and immune cells of the scar. The profile of scar-associated cluster 2 serves important functions by accurately describing the scar boundary at the cellular level.

The highest DEGs in Cluster 3 were collagen type I alpha 1 chain (Col1a1), collagen type I alpha 2 chain (Col1a2), insulin-like growth factor binding protein 6 (Igfbp6). and matrix gla protein. Col1a1 and Col1a2 belong to the type I collagen (Col I) family. To date, pericytes and fibroblasts have been reported to be the cell types that produce Col I after SCI [60, 61]. Among the ECM components, Col1a1 and Col1a2 have been reported to be the most strongly expressed in the injured spinal cord of the contusion SCI model at 14 days post-SCI (dpi).

Although ST (10× Visium) has many advantages, the biggest flaw is that the approach cannot achieve single-cell resolution at the current state of the technology. In the area of high cell density, each spot might include the transcriptome of a homogeneous or heterogeneous mixture of up to 15–20 cells. In order to identify the most likely cell types in different clusters, we used existing markers of different cell types in the spinal cord as gene sets and the AddModule Score function from the Seurat R package to evaluate the most likely cell types for each spot based on the scores. Then, the most likely cell type in each cluster was determined according to the overall score of all spots. Finally, the spatial maps of known typical markers in different cell types were used for verification. Taking advantage of these scores and marker validation, we confirmed that each cluster was the most likely reasonable cell type shown in the previous studies [10, 20, 21]. A possible explanation is that the scar architecture has a particularly layered pattern [9, 10, 13], such as a central core of fibrotic and inflammatory cells surrounded by microglia and astrocytes; this ultimately leads to each spot including the transcriptome of an homogeneous mixture of up to several cells. This means each spot might represent a group of several homogenous cells. Furthermore, we have verified the possible number for each spot at three stages, and found that each spot contained ~1–6 cells based on DAPI labeling. So, in the subsequent analysis we applied some tools that were used for scRNA-seq to analyze these spots. Fortunately, the cell divisions and spatiotemporal distributions revealed by these tools were consistent with the previous concepts [9, 10, 13, 20, 21, 62].

In order to further explore the function of these cell types, GO and KEGG pathway analysis of data was used to assess the enrichment for each cluster. The GO annotation matched well with the anatomical annotation (Fig. 1F). Specifically, some significant GO processes such as synaptic vesicle cycle, regulation of neurotransmitter levels, and ATP metabolic process were strongly associated with normal neuronal activities in Cluster 0. Some significant GO processes such as myelination, axon ensheathment, and positive regulation of neuron projection development were strongly associated with electrical signal transmission in Cluster 1. On the contrary, cluster 2 and cluster 3 cells exhibited damage-associated patterns of gene expression, with a significant enrichment in genes that function in extracellular structure organization, angiogenesis, wound healing, regulation of neuron death, regulation of inflammatory response, and regeneration (Fig. 1J).

Based on the symmetric distribution pattern of cluster 0 and cluster 1 cells in normal spinal cord, we divided the T10 right lateral spinal cord into four layers (Fig. S1F) to further explain the relations between the subgroups and spatiotemporal distributions. After counting all samples, we found that at least 1.2 mm from the scar center (~12 spots) covered the scar boundary. Subsequently, the GSVA scores for apoptosis, ferroptosis, GABAergic synapse, and PI_3_K-Akt signaling pathway were each counted (Fig. S1G). We found that the GSVA scores for ferroptosis and GABAergic synapse signaling pathway peaked in the scar center and were symmetrically distributed on the left and right at all three stages in layer 3 (Fig. 1K, L). Layer 3 enriched in cluster 0 neurons suffered from ferroptosis after SCI, consistent with previous studies [63]. Together, we present a spatiotemporal atlas that systematically describes the spatial archetypes and cellular heterogeneity of the glial scar in the early acute (3 dpi), subacute (7 dpi), and intermediate stages (14 and 28 dpi) after SCI [10, 20].

### Deciphering the Spatiotemporal Distribution of Resident Cells in the Scar

**Fig. 2:**
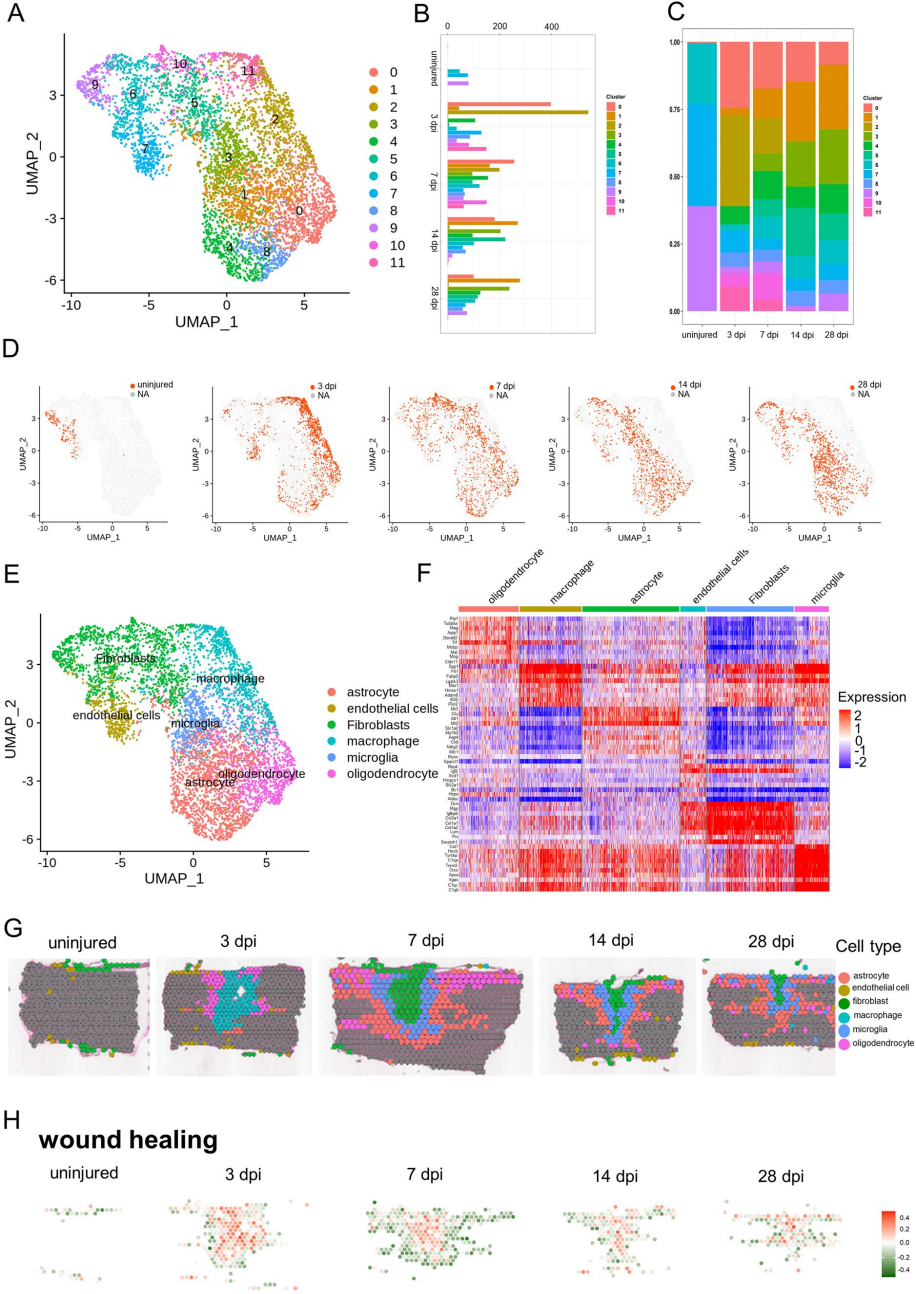
Cataloguing resident cells during glial scar maturation with ST. **A** UMAP spots showing the re-clustering analysis of cluster 2 cells and cluster 3 cells from four stages of scar maturation. **B** Histogram showing the number of spots in each subpopulation. **C** Bar plot showing the fraction of all spots comprising each subpopulation at different stages of scar maturation. **D** UMAP spots embedding overlay showing the distribution of spots at different time points after injury. **E** UMAP spots embedding overlay showing the six main cell clusters at different time points after injury. **F** Heat map showing the top 10 markers for the annotation of individual clusters shown as fraction of expression (color) of gene markers (columns). **G** Spatial maps showing the six main cell clusters at different time points after injury. **H** Spatial maps showing the GSVA scores of wound healing enriched in the six main cell clusters at different time points after injury.

To determine the heterogeneity within clusters 2 and 3 which represented multiple cellular components that comprise the glial scar, re-clustering analysis was applied and visualized on UMAPs (Fig. 2A), which revealed 12 clusters identified by annotated lineage markers (Fig. 2B, C). These 12 clusters represented virtually all major cell types that are known to comprise the scar: microglia, macrophages, astrocytes, oligodendrocytes, fibroblasts, and endothelial cells. From re-clustering the UMAPs at the different stages after SCI (Fig. 2D), we found that the cell spot distributions at 14 and 28 dpi were similar, and the distribution at 3 dpi was significantly different from those at 14 and 28 dpi. By contrast, the cell spots distribution at 7 dpi were observed between cell 3 dpi spots and 14 dpi spots. The cell spot distributions provided compelling evidence the division into time-windows: early acute (3 dpi), subacute (7 dpi), and intermediate stages (14 and 28 dpi) after SCI [1, 10, 20, 64–66].

To gain insights into the molecular basis of the injury responses mediated by these cell types, we divided all the re-clustered cell types into 6 distinct clusters: microglia, macrophages, astrocytes, oligodendrocytes, fibroblasts, and endothelial cells (Fig. 2E), consistent with previous concepts [3, 10, 20]. We found very few neurons in the scar area. A possible reason is that most damaged neurons were dead at the time-point of assessment [20, 21]. The transcriptional signatures are shown in Fig. 2F. We listed the strongly-expressed genes in each group in detail. For example, Decorin (Dcn) was especially expressed on fibroblasts; Plp1 was especially expressed on oligodendrocytes; and. Metallothionein 3 was especially expressed on astrocytes. By contrast, Spp1 was expressed on multiple cellular components (such as macrophages, fibroblasts, and microglia). Subsequently, these 6 cell clusters were mapped back to slices (Fig. 2G), and we found that macrophages first appeared in the scar area in the early acute stage (3 dpi), and then fibroblasts occupied the center of the scar in the subacute stage (7 dpi). From the center of the scar to the boundary, fibroblasts, microglia, astrocytes, and oligodendrocytes appeared in order at the subacute (7 dpi) and intermediate stages (14 and 28 dpi) after SCI, perfectly consistent with the previous concepts [3, 10, 20]. Furthermore, we used a novel gene set analysis method to infer activity maps by calculating the GSVA score of cell spots in the scar area [41]. Wound healing [3, 67], a selected GO term, was mapped in the scar region at three stages, and was significantly active (Fig. 2H). Taken together, our data revealed the spatiotemporal distribution of multiple cell types in the scar region and depicted the possible boundary of each cell type [3, 10, 20].

### Profiling Macrophage Infiltration of the Scar by ST Analysis

**Fig. 3:**
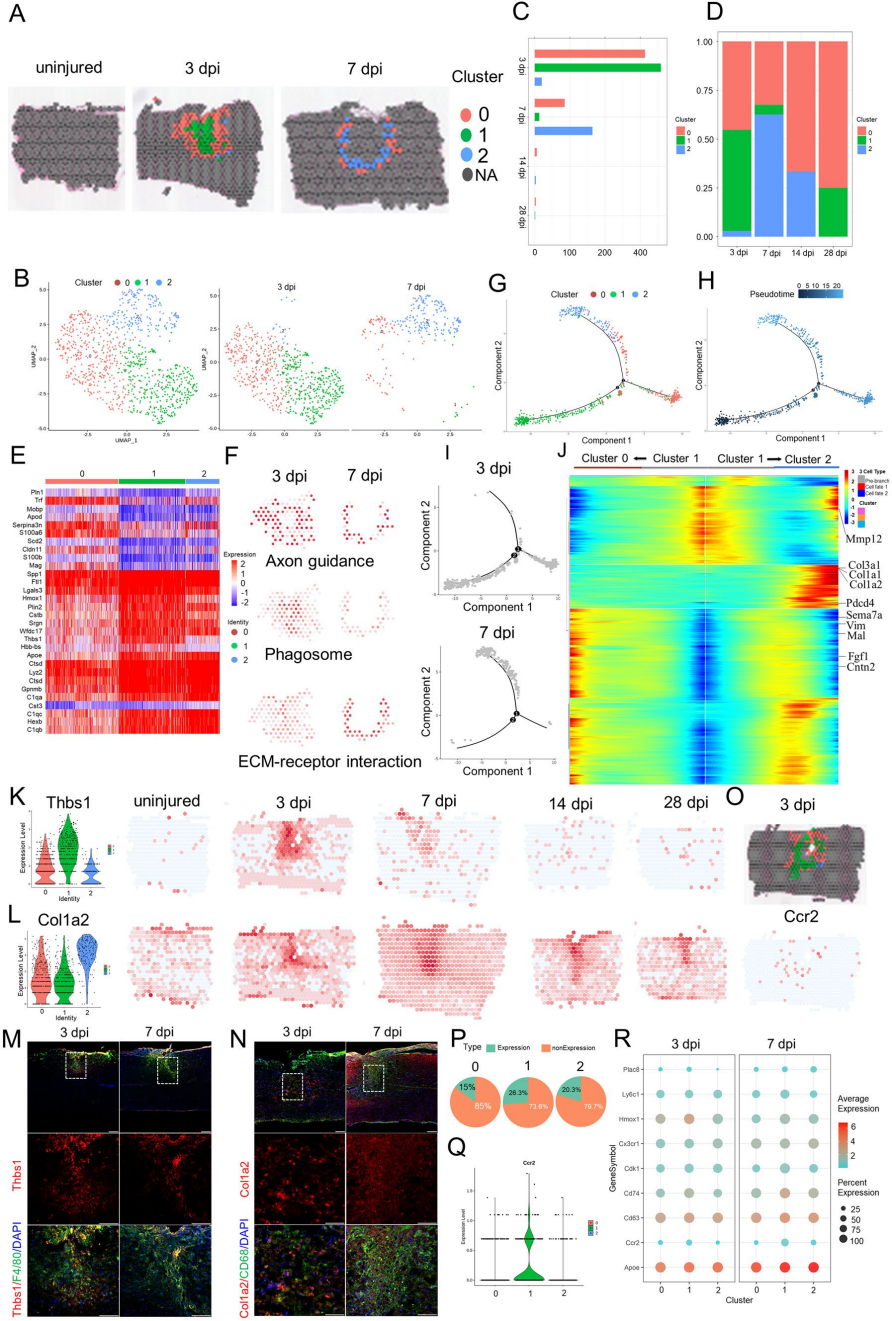
Phenotypic and functional heterogeneity of macrophage in the glial scar. **A** Spatial maps showing the distribution of three macrophage clusters in the glial scar at 3 and 7 dpi. **B** UMAP spots showing the three clusters of macrophages in the glial scar at 3 and 7 dpi. **C** Histogram showing the number of spots in each subpopulation at different time points after injury. **D** Bar plot showing the fraction of all spots comprising each subpopulation at different time points after injury. **E** Heat map showing the top 10 markers for the annotation of individual clusters shown as fraction of expression (color) of gene markers (columns). **F** Spatial maps showing the GSVA score of the axon guidance pathway enrichment in cluster 0 cells, phagosome pathway enrichment in cluster 1 cells and ECM-receptor interaction pathway enrichment in cluster 2 cells at different time points after injury. **G–I** Trajectory of macrophages in the glial scar from cluster 1 cells into clusters 0/2. **J** Heat map showing the gene expression dynamics along the trajectory. **K** Violin plots and spatial maps showing the expression of Thbs1 at different time points after injury. **L** Violin plots and spatial maps showing the expression of Col1a2 at different time points after injury. **M** Immunofluorescence (IF) in 3 dpi and 7 dpi scars stained for Thbs1 (red), F4/80 (green), and DAPI (blue) confirming that Thbs1 is overexpressed in the three macrophage clusters (*n =* 3; scale bar, 200 µm). **N** IF in 3 dpi and 7 dpi scars stained for Col1a2 (red), CD68 (green), and DAPI (blue) confirming that Col1a2 is overexpressed in the three macrophage clusters (*n =* 3; scale bar, 200 µm). **O** Spatial maps showing the distribution of the three macrophage clusters in the glial scar at 3 dpi and the expression of Ccr2 at 3 dpi. **P** Pie chart showing the percentages of Ccr2^+^ cells in the three macrophage clusters (*n =* 27). **Q** Violin Plots showing the expression of Ccr2 in the three macrophage clusters (*n =* 27). **R** Dot plots showing the marker genes that best identify each cell type.

To determine the heterogeneity within the macrophage population, re-clustering analysis was applied and visualized on spatiotemporal maps (Fig. 3A, B). We found three macrophage subtypes, which were labeled clusters 0 to 2. Cluster 0 was identified by strong expression of lysozyme (Lyz2), a specific marker for macrophages [20], and thyrotropin releasing hormone. Cluster 0 comprised 45.3% of the macrophages present at 3 dpi (Fig. 3C, D). Cluster 1 expressed higher levels of platelet factor 4 (Pf4) (a specific marker for macrophages) and the anti-inflammatory marker arginase 1 (Arg1) [20] than Clusters 0 and 2 (Figs 3E, S2A), and comprised 51.8% of the macrophages present at 3 dpi. Cluster 2 expressed a moderate level of Lyz2 and a low level of Pf4, and comprised 2.9% of the macrophages present at 3 dpi (Figs 3D, S2A).

Although these three subtypes did not precisely correspond to the traditional M1/M2 division of macrophage subsets, they were consistent with the previous macrophage division in SCI [20]. Cluster 1 cells expressed higher levels of Arg1 and CD14 than Clusters 0 and 2 (Fig. S2A), indicating that Cluster 1 cells were likely to be M2 macrophages. Interestingly, ST maps showed that the macrophages of Cluster 1 cells were always in the central part of the injury site and surrounded by the macrophages of Cluster 0. The macrophages of Cluster 2 were always in the periphery of the injury site at 3 dpi (Fig. 3A). Subsequently, cluster 1 cells decreased dramatically and cluster 2 cells significantly increased. Interesting, cluster 2 cells interlaced with cluster 0 cells and formed a circular band of cells at 7 dpi. These three clusters became rare at 14 and 28 dpi. In addition, ST maps showed that expression of the macrophage marker Pf4 was significantly upregulated at 3 and 7 dpi, and then returned to baseline at the intermediate stage (Fig. S2A). There was a clear shift in the expression of the M2 macrophage marker Arg1, which peaked at 3 dpi and then fell rapidly. This is consistent with the change of macrophage phenotype after injury [68]. The Pf4^+^ cells residing in the scar at the intermediate stage (14 and 28 dpi) mainly represented M1 macrophages, which resulted in a microenvironment in the scar hostile to regeneration, and they were also characterized by CD86 and CD32 maps (Fig. S2A). This is the first spatiotemporal distribution of macrophage subsets in the scar based on ST.

It is traditionally believed that M2 macrophages promote CNS repair while limiting secondary inflammation-mediated injury [68]. We found the macrophages of cluster 1 cells not only decreased dramatically, but were also surrounded by the macrophages of clusters 0 and 2. To investigate the functions of these clusters, we listed their top 10 marker genes (Fig. 3E), and the enriched GO terms based on the strongly expressed genes (Fig. S2B). The top terms showed Cluster 1 cells indeed had a high enrichment factor in wound healing. However, Cluster 1 cells also exhibited unique patterns of gene expression, with significant enrichment for chemotaxis, regulation of inflammatory response, response to interferon-gamma, and cellular response to interleukin-1. Interestingly, cluster 0 cells were associated with border and exhibited significant enrichment for axonogenesis, axon guidance, axon extension, and axon ensheathment. It seems that they might promote axon regeneration into the scar from both sides. The impressive functions matched the unique spatiotemporal distributions of cluster 0 cells that resided in the scar periphery, which allowed cluster 0 cells to have better access to axon terminals. The GSVA score of axon guidance pathway analysis also supported this view (Fig. 3F). In addition, the top 4 GO terms exhibited by cluster 2 cells were extracellular structure organization, extracellular matrix organization, positive regulation of cell migration, and angiogenesis. Particularly, the GO term negative regulation of immune system process matched the traditional functions of M2 macrophages, limiting secondary inflammation-mediated injury [68]. So, we hypothesized that cluster 2 cells represent the continuators of the cluster 1 cells.

To test this hypothesis, we conducted the trajectory analysis of the three subtypes (Figs 3G–J, S2C). Two branches were built (Fig. 3G) and then the pseudo-time trajectory algorithm was applied (Fig. 3H). We found the evolutionary trajectory and transcriptional connections among the subtypes. The results indicated that the trajectory direction started from cluster 1 cells to a separate branch consisting of cluster 0 cells and cluster 2 cells (Fig. 3H, I). We found that the cell fate 1 branch was enriched for genes associated with axon ensheathment, such as Sema7a, Vim, Mal, Cntn2, and Fgf1, while the cell fate 2 branch showed increased expression of extracellular matrix organization and negative regulation of immune system process-related genes, such as Gpnmb, Mmp12, Col3a1, Col1a1, and Col1a2 (Fig. 3J).

The DEGs provided a molecular signature for each cell type, and they were different from the canonical markers (Table S3). For example, the highest DEGs in Cluster 1 were non-canonical genes such as Thbs1 (thrombospondin 1) and Plin2 (perilipin 2). Thbs1 signaling has been implicated in the acute neuropathological events that occur in spinal cord microvascular endothelial cells following SCI [69]. In addition, unsatisfied intrinsic neurite growth capacity results in significant obstacles for injured spinal cord repair. Thbs1 has been reported to be a neurite outgrowth-promoting molecule. Overexpression of Thbs1 in bone marrow mesenchymal stem cells promotes neurite outgrowth of motor neurons exposed to oxygen-glucose deprivation and rat models of SCI *in vitro* and *in vivo* [70]. ST maps showed that the expression of Thbs1 was significantly upregulated at 3 dpi and 7 dpi, and then returned to baseline (Figs 3K, S2C). Immunostaining showed that the majority of Thbs1^+^ cells were P4/60 (a macrophage marker)-positive at 3 dpi and 7 dpi (Fig. 3M). Although Spp1 was the highest DEG in Cluster 1, it was expressed in multiple cell types and was unfit as a marker of non-canonical macrophages (Fig. S1E).

Another unexpected finding is that the macrophages in Cluster 2 expressed higher levels of Col I, including Col1a2, Col1a1, and Col3a1, which is different from the previous study that fibroblasts and pericytes produce Col I after SCI [71]. Col I was strongly expressed in the spinal cord, consistent with previous findings. Specifically, Col I induces astrocytic scar formation *via* the integrin-N-cadherin pathway during the scar-forming phase [71]. This is consistent with GO terms associated with extracellular matrix organization. ST maps showed that the expression of Col1a2 was rare in the intact spinal cord, and was markedly upregulated after injury. It peaked at 7 dpi and was maintained at high level for 28 days (Fig. 3L). Immunostaining showed that the majority of Col1a2^+^ cells are CD68 (a macrophage marker)-positive at 3 dpi (Fig. 3N). In addition, Msr1 (macrophage scavenger receptor 1) has been implicated in many macrophage-associated physiological and pathological processes including atherosclerosis, Alzheimer's disease, and host defense. In the scar, Msr1 promotes the formation of foam macrophages and neuronal apoptosis after SCI [72]. ST maps showed that Msr1^+^ cells significantly increased at 3 dpi and 7 dpi in the injury site (Fig. S2D). Activation of the innate immune system promotes regenerative neurogenesis in zebrafish. TNFα from pro-regenerative macrophages induces Tnfrsf1a-mediated AP-1 activity in progenitors to increase the regeneration-promoting expression of hdac1 and neurogenesis. But it is unknown whether mammals retain this mechanism. Our ST maps showed that at least Tnfrsf1a was widespread at 3 dpi and 7 dpi in the scar (Fig. S2E) [73].

In terms of macrophage origin, the traditional view is that bone marrow-derived mononuclear macrophages (also known as recruited macrophages) are recruited into damage tissue *via* chemokine (C-C motif) receptor 2 (Ccr2) in response to chemokines and inflammatory signals and affect wound healing by secreting inflammatory factors such as TNFα [68, 74]. Ccr2 encodes a receptor for monocyte chemoattractant protein-1, a chemokine that specifically mediates monocyte chemotaxis, and is expressed on monocytes and macrophages, but not in microglia or tissue-resident macrophages in the resting state [75]. A recent study found the mouse meninges host a rich repertoire of immune cells mediating CNS immune surveillance and are supplied not from the blood but by adjacent skull and vertebral bone marrow. In SCI, local bone marrow-derived monocytes infiltrate the spinal cord from the dural meninges [76]. We set out to distinguish the location and proportion of macrophages from the blood and tissue-resident macrophages from adjacent dural meninges. ST maps showed that Ccr2^+^ monocyte-derived macrophages were present in the scar, but their proportion in the injury site was low (Fig. 3O, P), consistent with previous studies. Violin plots showed that the expression of Ccr2 was low in these three clusters (Fig. 3Q). Last, we profiled the combination of the activated macrophage markers to distinguish these clusters, and found that cluster 1 cells expressed higher levels of heme oxygenase 1 at 3 dpi. In summary, our data revealed the spatiotemporal distribution of macrophage subtypes at the injury site after SCI.

### ST Analysis Reveals Spatiotemporal Changes in Fibroblasts

**Fig. 4:**
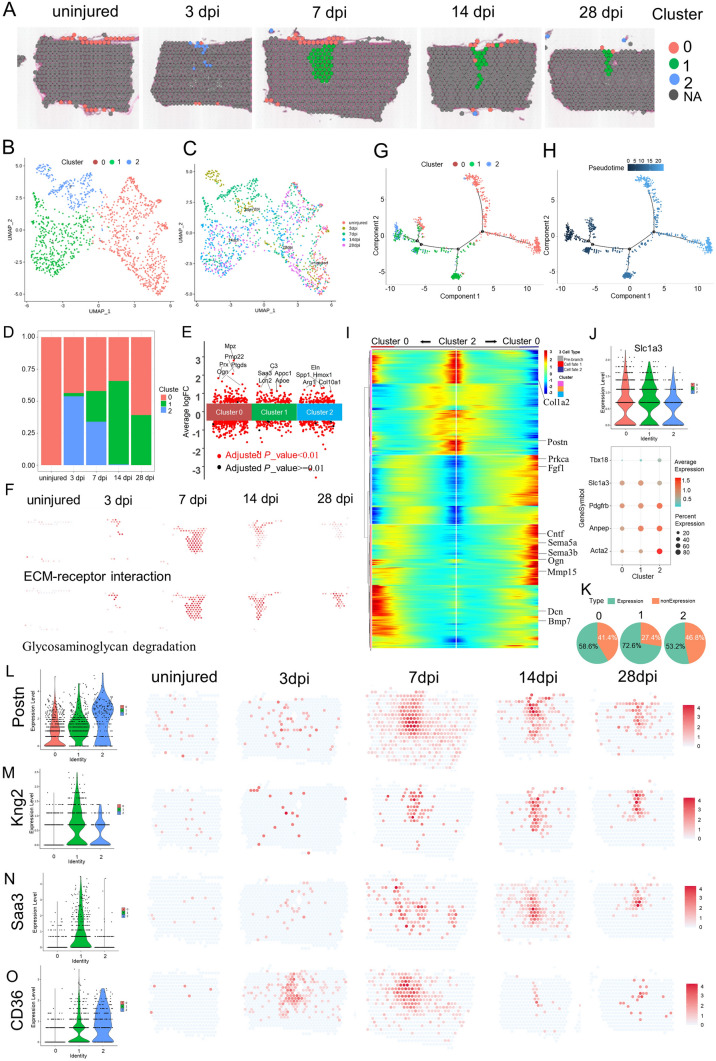
Phenotypic and functional heterogeneity of fibroblasts in the glial scar. **A** Spatial maps showing the distribution of the three fibroblast clusters in the glial scar at different time points after injury. **B** UMAP spots showing the three clusters of fibroblasts in the glial scar. **C** UMAP spots embedding overlay showing the distribution of spots at different time points after injury. **D** Bar plot showing the fraction of all spots comprising each subpopulation at different time points after injury. **E** Jitterplot showing the top 5 differentially-expressed genes. **F** Spatial maps showing the GSVA scores of the ECM-receptor interaction pathway enrichment in cluster 2 cells and the glycosaminoglycan degradation pathway enrichment in cluster 1 cells at different time points after injury. **G**, **H** Trajectory of fibroblasts in the glial scar. **I** Heat map showing the upregulated or downregulated genes in different cell fates. **J** Violin plots showing the expression of Slc1a3 in the three clusters. **K** Dot plots showing the marker genes that best identify type A pericytes. **L** Pie chart showing the percentages of Slc1a3^+^ cells in the three fibroblast clusters (*n =* 32). **M** Violin plots and spatial maps showing the expression of Postn, a myofibroblast differentiation marker, at different time points after injury. **N** Violin plots and spatial maps showing the expression of Kng2 at different time points after injury. **O** Violin plots and spatial maps showing the expression of Saa3, an inflammatory ligand, at different time points after injury.

Fibroblasts are the main producers of the matrix (including the ECM) and constitute the basic framework of tissues and organs [77]. To assess the heterogeneity among fibroblasts at the injury site, re-clustering analysis was performed and visualized on spatiotemporal maps (Fig. 4A) and a UMAP (Fig. 4B, C). We identified three fibroblast subtypes, which were labeled clusters 0 to 2. Based on the spatiotemporal maps, we found that cluster 2 mainly appeared in the scar at 3 dpi. Cluster 1 appeared in the scar at 7 dpi and was located in the center of scar. On the contrary, cluster 0 was located in the meninges of the spinal cord. The fibroblast number in Cluster 1 peaked at 14 dpi (Fig. 4D). Cluster 0 was identified by high expression of prostaglandin D2 synthase (Ptgds) and Igfbp6 (Fig. 4E). Ptgds is preferentially expressed in the brain and functions as a neuromodulator as well as a trophic factor in the CNS. In the chronic constriction injury of the sciatic nerve model, Ptgds is overexpressed in the lumbar spinal cord, but not in the striatum [78]. Igfbp6, a member of the insulin-like growth factor-binding protein family, modulates insulin-like growth factor activity. In the spinal cord, meningeal cells, interneurons in the deep part of the dorsal horn and around the central canal, and motoneurons are Igfbp6-positive [79]. Igfbp6 plays a key role in neuronal apoptosis after SCI. In the acute SCI model, Igfbp6 is significantly upregulated and is co-localized with active caspase-3 and p53 in neurons. When Igfbp6 is knocked down, the protein levels of active caspase-3 and Bax, as well as the number of apoptotic primary neurons, are significantly decreased [80]. ST maps showed that Igfbp6 and Ptgds were strongly expressed in Cluster 0 cells (Fig. S3A).

To investigate the functions of these clusters, we listed the top 5 DEGs between them (Fig. 4E), and assessed the GO term enrichment based on highly-expressed genes (Fig. S3B). The top terms showed that cluster 2 cells were highly enriched in wound healing. In addition, cluster 2 cells also exhibited significant enrichment for angiogenesis, extracellular matrix organization, response to transforming growth factor beta, and collagen metabolic process. The GSVA score of ECM-receptor interaction pathway analysis also supported this view (Fig. 4F). But cluster 2 cells gradually diminished. Interestingly, cluster 0 cells were associated with meninges and exhibited significant enrichment for axonogenesis, axon development, axon guidance, axon ensheathment, and regulation of epithelial cell proliferation. What is more, the top 5 GO terms exhibited by cluster 1 cells were leukocyte chemotaxis, phagocytosis, regulation of immune effector process, T cell activation, and activation of immune response. Cluster 1 cells gradually became the main component in the scar and their enrichment was associated with inflammatory hyperactivation in the intermediate stage after SCI, consistent with previous studies [81]. The GSVA score of glycosaminoglycan degradation in the top 10 pathway analysis enrichment in cluster 1 cells significantly increased from 7 dpi to 28 dpi (Fig. 4F), which may be related to the maturation and contracture of the scar, resulting in compartmental structures containing multiple cells (Fig. S3C).

We applied trajectory analysis to verify the evolutionary relationship between these three cell populations (Fig. 4G–I). Two branches were found (Fig. 4G) and then the pseudo-time trajectory algorithm was applied (Fig. 4H). The trajectory direction started from cluster 2 cells and cluster 0 cells to a separate branch consisting of cluster 0 (Fig. 4H, I). We found that the cell fate 1 branch showed increased expression of extracellular matrix organization, such as Dcn, Col1a2, and Bmp7, while the cell fate 2 branch was enriched for genes associated with axon guidance and axonogenesis, such as Sema3b, Sema5a, Periostin (Postn), Cntf, Ogn, Mmp15, and Fgf1 (Fig. 4I). Interesting, the origin of cluster 1 cells came from three directions. It was clearly evident that a certain percentage of cluster 1 cells came from adjacent to the dural meninges after SCI. Our data showed that cluster 2 cells appeared at 3 dpi and transformed into cluster 1 cells. Where did these cells originate? Previous studies have reported that, in mouse models that develop fibrotic tissue, the primary source of scar-forming fibroblasts is type A pericytes. Perivascular cells with a type A pericyte marker profile also occur in the human brain and spinal cord, suggesting it is conserved across diverse CNS lesions [61, 82]. Attenuation of pericyte-derived fibrosis is a promising therapeutic approach to facilitate recovery following SCI [83]. We hypothesized that cluster 2 cells originate from type A pericytes and then tested the expression of their marker GLAST (gene name Slc1a3). We assessed the expression level of GLAST and the proportions of GLAST^+^ cells in the three clusters, and found GLAST^+^ cells were the primary origin of fibroblasts in the scar (Fig. 4J, K), consistent with the previous study [61].

Homeostatic fibroblasts in cluster 0 were identified based on their spatiotemporal distribution and the expression of several annotated markers of steady-state fibroblasts, such as P4hb and Gsn. These fibroblasts in cluster 0 were the predominant subtype in the uninjured spinal cord, but by 3 dpi they were replaced by the damaged-associated fibroblasts (DAFs) of clusters 1 and 2 (Figs 4D, S3A). DAFs were always in the center of the scar. DAFs were identified by high expression of Gpnmb, Ftl1, Col3a1, and Postn [20]. The myofibroblast differentiation marker Postn is an important extracellular matrix protein that coordinates a variety of cellular processes and functions in tissue development and regeneration, including wound healing and ventricular remodeling following myocardial infarction [84]. Upregulation of Postn has been reported in different diseases characterized by oxidative stress and inflammatory responses [20, 85, 86]. In addition, upregulation of Postn suppresses SLC7A11 expression through the inhibition of p53 in VSMCs and increases the sensitivity of cells to ferroptosis. More importantly, Postn can remodel the injury environment and has been identified as a therapeutic agent for traumatic injury of the CNS. Astroglia-derived Postn promotes axonal regeneration through the focal adhesion kinase-Akt signaling pathway after SCI [87]. But Postn has also been reported to be a key player in scar formation after traumatic SCI. Genetic deletion of Postn in mice reduces scar formation at the lesion site by inhibiting the proliferation of pericytes. Moreover, the pharmacological blockade of Postn restrains scar formation and improves the long-term functional outcome after SCI [88]. Conversely, our ST maps found the expression of Postn predominantly in the DAFs of clusters 1 and 2. Although the expression of Postn also peaked at 7 dpi, it was maintained at a high level of expression until 28 dpi (Fig. 4L). We also found that non-canonical genes such as kininogen 2 (Kng2) (Fig. 4M) and serum amyloid A3 (Saa3) displayed better specificity than canonical fibroblast markers such as P4HB, which was expressed in multiple cell types (Fig. S3A). The expression of Saa3 in cluster 1 increase gradually and reached the highest level at 7 dpi (Fig. 4N). Saa3 is a pseudogene, and acts as an inflammatory ligand and target of the long non-coding RNA metastasis-associated lung adenocarcinoma transcript 1. Saa3 was accompanied by an increase in the inflammatory mediators TNFα and interleukin 6 [89]. In addition, Saa3 is a key mediator of the pro-tumor genic properties of cancer-associated fibroblasts in pancreatic tumors [90]. So far, the role of Saa3 in scar formation is unclear.

**Fig. 5:**
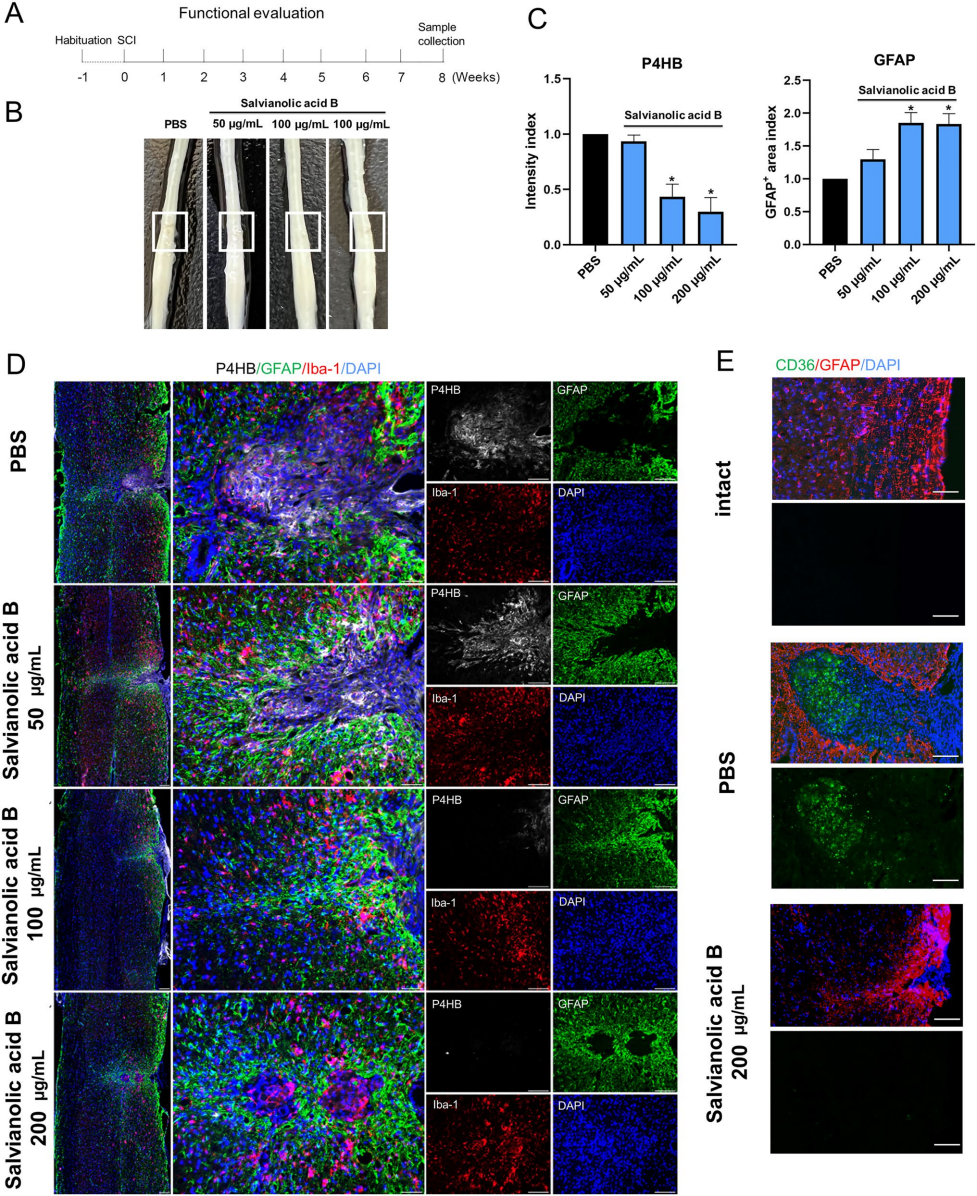
Pharmacological blockade of CD36 restrains fibrosis. **A** Experimental workflow and analysis for SAB treatment after T10 right lateral hemisection. **B** Representative images of injured mouse spinal cords treated with PBS and with SAB (50, 100, and 200 µg/mL) at 8 weeks post-infarction. The injury site is boxed in white. **C, D** SAB treatment reduces accumulation of P4HB^+^ cells in the scar core and promotes GFAP^+^ cells crossing the scar core and forming bridges in mice treated with SAB (200 µg/mL) (scale bar, 200 µm). **E** SAB treatment reduces the expression of CD36 in the scar core (scale bar, 100 µm).

In the spinal cord, a fibrotic scar gradually matures (Fig. S3C) and limits CNS regeneration in adult mammals. Similarly, skin wounds generally heal by scarring, a fibrotic process mediated by the Engrailed-1 (En1) fibroblast lineage [91]. Preventing En1 activation in fibroblasts yields wound regeneration without scarring. It is unknown whether fibrotic scar-formation in the spinal cord needs En1 activation. In the current study, ST maps showed that En1 was rarely expressed in the spinal cord before and after injury (Fig. S3D), suggesting that the CNS has a different mechanism of fibrotic scar-formation. Another study found that Jun promotes hypertrophic skin scarring *via* CD36 in *in vitro* and *in vivo* models [45]. Indeed, we found that injury enhanced Jun expression (Fig. S3D). Particularly, CD36 was evidently induced in the injury and tightly limited to the area of the scar (Fig. 4O). A previous study has shown that CD36 deletion improves recovery from SCI in the mouse, but they did not identify the cell type expressing it [92]. In our data, there was a fair amount of overlap between CD36^+^ spots and a-SMA^+^ (gene name Acta2)-activated fibroblasts (Fig. S3A), suggesting that CD36^+^ is involved in contractures of activated fibroblasts for scar formation. Specifically, 59.83% of CD36^+^ cells were GLAST-positive at 28 dpi. Since targeting CD36 may be a direction for spinal scar therapy, we use SAB, a blood–brain barrier permeant and CD36 inhibitor [45], to reduce the activated fibroblasts (Fig. 5A, B). Although the BMS assessment of motor function in injured mice treated with PBS and with SAB (50,100, and 200 µg/mL) showed no difference at the time of observation because of the rapid recovery of motor function after T10 right lateral hemisection, the injury site showed a reduced accumulation of P4HB^+^ cells in the scar core (Fig. 5C, D). Furthermore, GFAP^+^ (an established marker for activated astrocytes) cells crossed the scar core of mice treated with SAB (200 µg/mL) and formed bridges, whereas GFAP^+^ cells were absent in the scar core of mice treated with PBS (Fig. 5D). In addition, SAB (200 µg/mL) significantly inhibited the CD36 expression induced by SCI in activated fibroblasts (Fig. 5E). Taken together, we described the spatiotemporal distribution and origin of fibroblast subtypes at the injury site after SCI. Furthermore, the distribution of CD36 in ST maps inspired us to find a strategy to remedy fibrosis.

### ST Analysis of Microglial Heterogeneity Reveals their Specific Roles During Gliosis

**Fig. 6:**
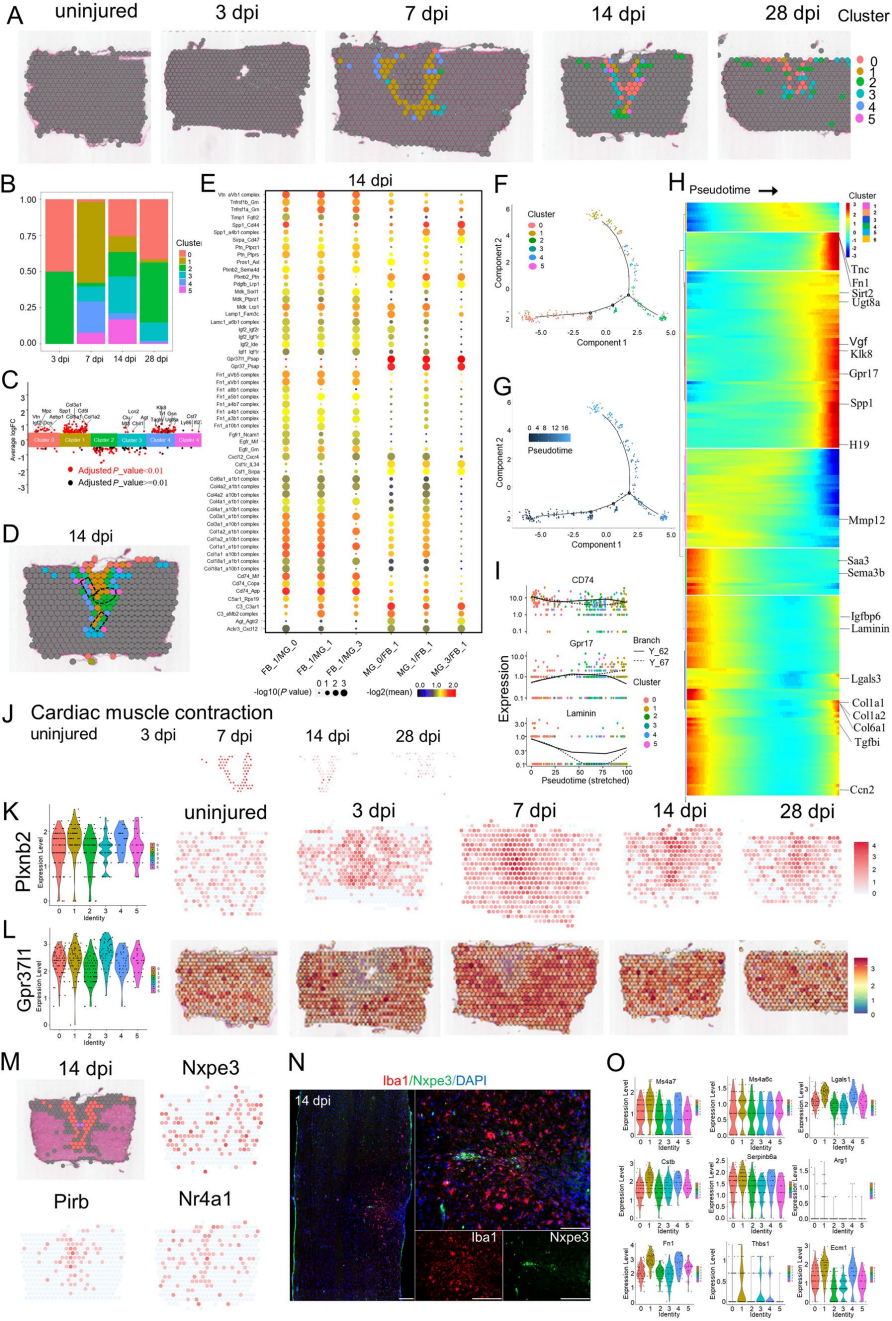
Phenotypic and functional heterogeneity of microglia in the glial scar. **A** Spatial maps showing the distribution of 6 microglia clusters in the glial scar at different time points after injury. **B** Bar plot showing the fraction of all spots comprising each subpopulation at different time points after injury. **C** Jitterplot showing the top 5 differentially-expressed genes (DEGs) in each subpopulation. **D** Definition of the boundary areas to study the interaction between two neighboring microglia clusters in the scar. Regions 2 spots wide along the boundary lines in each cluster are selected. **E** Dot plot showing the mean interaction scores between neighboring clusters at the boundaries for ligand-receptor pairs. The ligand-receptor pairs are listed on the left. The size of a circle denotes the p-value, and the color denotes the mean interaction score. **F, G** Trajectory of microglia in the glial scar. **H** Heat map showing the changes in genes expression along a spatial trajectory. **I** Correlation of the expression of CD74, Gpr17, and laminin with pseudotime. **J** Spatial maps showing the GSVA score of the cardiac muscle contraction pathway enrichment in cluster 1 cells at different time points after injury. **K** Violin plots and spatial maps showing the expression of Plxnb2 at different time points after injury. **L** Violin plots and spatial maps showing the expression of Gpr37l1 at different time points after injury. **M** Spatial maps showing the expression of selected DEGs in spots that cross the fibroblast scar and form bridges at 14 dpi. **N** IF-stained 14-dpi scars for Nxpe3 (red) and DAPI (blue) (scale bars, 200 µm). **O** Violin plots showing the expression of selected genes contributing to microglia-organized scar-free spinal cord repair in neonatal mice.

Microglia play a crucial part in scar formation [77, 93]**.** To assess the cellular heterogeneity among microglia at the injury site, re-clustering analysis was performed and visualized on spatiotemporal maps (Fig. 6A) and a UMAP (Fig. S4A, B). We identified six microglial subtypes, which were labeled clusters 0 to 5 (Figs 6B, Fig. S4C). Based on the spatiotemporal maps, we found that cluster 1 cells were mainly distributed in the boundary of fibroblast foci in the scar at 7 dpi and then diminished rapidly. On the contrary, cluster 0 cells replaced cluster 1 cells in the boundary of fibroblast foci. The number of microglia in Cluster 0 increased gradually from 7bdpi to 28bdpi (Fig. 6B). ST maps characterized the expression of P2Y12, a marker for homeostatic microglia, as well as CD68 and Iba1, markers for activated microglia (Fig. S4D).

To characterize the functions of these clusters, we listed top 5 DEGs for each cluster (Fig. 6C), and the enriched GO terms based on highly-expressed genes (Fig. S4E). The top terms showed that cluster 0 cells were highly enriched in axon ensheathment, axon development, and ensheathment of neurons. The pathway of ECM–receptor interaction was also significantly enriched in cluster 0 cells. Interestingly, ST maps showed that a minority of cluster 0 cells crossed the boundary of the fibroblast scar. Clusters 1, 2, and 3 cells exhibited significant enrichments for angiogenesis, extracellular matrix organization, and extracellular structure organization. The GSVA score for cardiac muscle contraction pathway analysis peaked at 7 dpi and might explained the phenomenon of scar contracture (Fig. 6J). In addition, cluster 5 cells exhibited significant enrichment in the negative regulation of cell motility, cell migration, locomotion, and immune system process, which are similar to the functions of neonatal microglia [93]. Unfortunately, cluster 5 cells were few, and were not sufficient to change the whole inflammatory environment.

To next investigate the communication and interaction between microglia and fibroblasts, the interface regions of clusters were selected with the range of at least 2 spots for each cluster. The top enriched gene-pairs included Tnfrsf1b_Grn, Tnfrsf1a_Grn, Spp1_CD44, Plxnb2_Ptn, Gpr37l1_Psap, and Gpr37_Psap (Figs 6D, E, S4F, G). For example, Plxnb2 is a transmembrane receptor that participates in axon guidance and cell migration in response to semaphorins [94]. Plxnb2 is induced in injury-activated microglia and macrophages early after SCI and facilitates the formation of concentric rings of microglia and astrocytes around the necrotic core of the lesion in a mouse model of SCI [95]. ST maps showed Plexnb2^+^ cells enriched in the scar area (Fig. 6K). In addition, Gpr37l1_Psap and Gpr37_Psap were the top 2 enriched gene-pairs between microglia clusters 0/1/3 and fibroblast cluster 1 from 14 dpi to 28 dpi. Prosaposin (gene name PSAP) is secreted by various cell types in response to cellular stress. Secreted PSAP initiate endocytosis or pro-survival signaling pathways *via* binding to Gpr37 and Gpr37l1 [96, 97]. ST maps showed that PSAP was enriched in scar-resident fibroblasts and Gpr37l1 was enriched in scar-resident microglia (Fig. 6L), but the exact role of the PSAP secreted by fibroblasts awaits future exploration.

Subsequently, we conducted trajectory analysis (Fig. 6F–I) and applied the pseudo-time trajectory algorithm (Fig. 6G). The trajectory direction started from cluster 1 cells and cluster 0 cells to a separate branch consisting of clusters 2–5 (Fig. 6H). We found that the expression level of enriched genes associated with axon ensheathment and axon development, such as Tnc, Fn1, Sirt2, Ugt8a, and Klk8, gradually increased following scar stabilization. On the contrary, Sema3b, Mmp12, and Laminin gradually declined. We also noted that the expression level of G protein-coupled receptor 17 (Gpr17) significantly increased (Fig. 6H,I). Gpr17, a P2Y-like receptor, may act as a sensor of damage that is known to be activated by both uracil nucleotides and cysteinyl-leukotriene released in the lesioned area, and could also participate in post-injury responses [98], In non-injured spinal cord parenchyma, Gpr17 is present on a subset of neurons and oligodendrocytes at different stages of maturation, but it is not expressed by astrocytes. Induction of SCI results in marked cell death of Gpr17^+^ neurons and oligodendrocytes inside the lesion followed by the appearance of proliferating Gpr17^+^ microglia/macrophages migrating to and infiltrating into the lesioned area [99]. In vivo pharmacological or biotechnological knockdown of Gpr17 markedly prevents the evolution of brain infarcts, suggesting that Gpr17 is a mediator of neuronal death at this early ischemic stage [100]. Gpr17 also acts as an intrinsic timer of oligodendrocyte differentiation and myelination. Pharmacological targeting of Gpr17 signaling in OPCs and microglial inhibition of oligodendrocyte maturation together promote the robust myelination of regenerated axons after CNS injury [101]. The classically-activated neuroinflammatory microglia induce A1 astrocytes by secreting Il-1α, TNF, and C1q, and these cytokines together are necessary and sufficient to induce A1 astrocytes [62]. ST maps characterized the enhanced expression of C1qa in 6 microglial subtypes at different time point (Fig. S4H).

A recent study found microglia-organized scar-free spinal cord repair in neonatal mice. Fibronectin^+^ microglia in cluster 3 (MG3) formed bridges between the two stumps starting from 3 dpi. Activated microglia were observed inside the lesion in the absence of GFAP^+^ astrocytes and collagen I^+^ fibroblasts [93]. In our adult mouse model, ST maps showed A few microglia in cluster 0 crossed the fibroblast scar and formed bridges between the two stumps at 14 dpi. Unfortunately, the bridges eventually disappeared at 28 dpi. We profiled selected cells that crossed the fibroblast scar and found that Nxpe3, Pirb, and Nr4a1 were specifically and strongly expressed in these cells (Fig. 6M). Immunohistochemistry verified the expression of Nxpe3 in the boundary of the fibroblast scar (Fig. 6N). Nxpe3, a highly conserved neurexophilin and PC-esterase (NXPE) central domain, binds α-neurexins and promotes adhesion between dendrites and axons [102]. Finally, we profiled ECM-related genes (such as Fn1, ECM1, and Thbs1), wound-healing genes (such as Anxa1), positive regulation of cell adhesion (such as Spp1), negative regulation of hydrolase activity (such as Cstb, Serpinb6a, and Stfa1), negative regulation of immune system process (such as Arg1), disease-associated genes (such as Igf1 and Clec7a), embryonic stem cell-related genes (such as Ms4a7, Lgals, and Ms4a6c), and phospholipase inhibitor activity (such as Anxa2, Anxa5, and Apoc1) in 6 microglia subtypes. These genes were highly expressed in MG3. But we found that Stfa1 and Clec7a were not expressed in the 6 microglia subtypes, and the expression levels of Arg1 and Thbs1 were very low (Fig. 6O). Specifically, Anxa1, a potent anti-inflammatory protein, may contribute to the ability of MG3 cells to mediate the rapid of resolution inflammation and were barely expressed in the microglia of clusters 2 and 3, the two major types of microglia in the scar at 28 dpi (Figs S4I, 6B). These differences between the microglia in the 6 clusters with MG3 cells in neonatal mice might contribute to deciphering the difference in regenerative capacity between adult and neonatal mice.

### Six Different Astrocyte Subtypes are Identified by ST

**Fig. 7:**
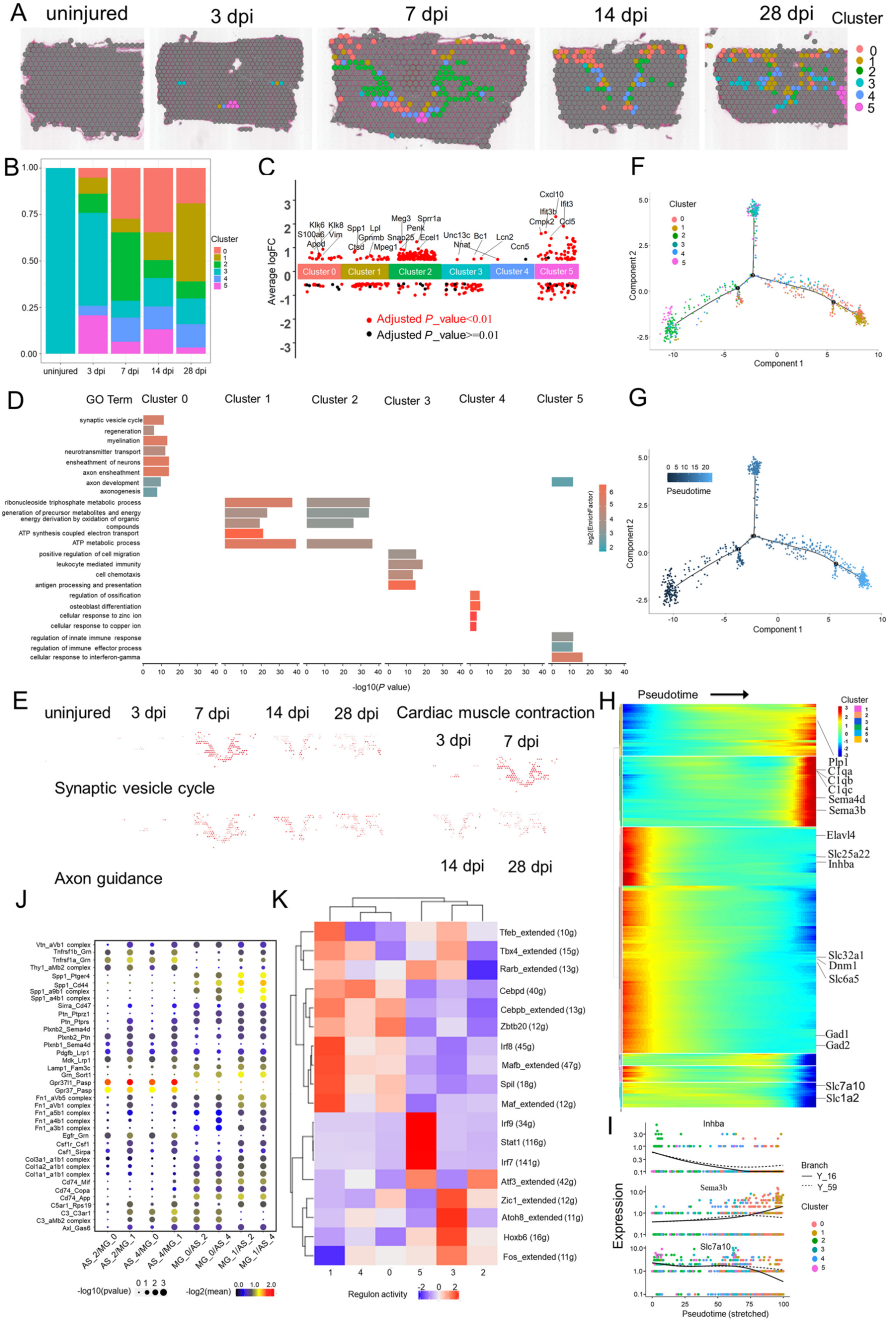
Phenotypic and functional heterogeneity of astrocytes in the glial scar. **A** Spatial maps showing the distribution of 6 astrocyte clusters in the glial scar at different time points after injury. **B** Bar plot showing the fraction of all spots comprising each subpopulation at different time points after injury. **C** Jitterplot showing the top 5 differentially expressed genes in each subpopulation. **D** Selected GO terms that were enriched for each cluster using Fisher’s exact test. **E** Spatial maps showing the GSVA score of the synaptic vesicle cycle and axon guidance pathways enriched in cluster 0 cells, and the cardiac muscle contraction pathway enriched in cluster 1 cells at different time points after injury. **F**, **G** Trajectory of astrocyte in the glial scar. **H** Heat map showing the changes in gene expression along a spatial trajectory. **I** Expression of Inhba, Sema3b, and Slc7a10 correlates with pseudotime. **J** Dot plot showing the mean interaction scores between neighboring clusters at the boundaries for ligand-receptor pairs. The ligand-receptor pairs are listed on the left. The size of a circle denotes the p-value and the color denotes the mean interaction score. **K** Heat map showing the enriched transcription factors by single-cell regulatory network inference and clustering analysis in different clusters after injury.

Astrocytes are thought to be the main drivers of encirclement. Tightly-connected astrocytes continuously reshape the boundary at the edge of an injury, wrapping immune cells and fibroblast-like cells with ephrin-mediated cell adhesion and spatially isolating the remaining neural tissue from injury and fibrosis [3, 81, 103]. We next assessed the cellular heterogeneity among astrocytes at the scar, using re-clustering analysis and visualization on spatiotemporal maps (Fig. 7A) and a UMAP (Fig. S5A, B). We identified 6 astrocyte subtypes, labeled clusters 0 to 5 (Figs 7B, S5C). Based on the spatiotemporal maps, we found cluster 2 cells mainly were distributed in the grey matter layer of concentric rings of microglia in the scar at 7 dpi and diminished rapidly; these cells might represent protoplasmic astrocytes. On the contrary, cluster 0 cells were mainly distributed in the white matter layer of the scar, suggesting that these cells represent fibrous astrocytes. The number of astrocytes in Cluster 0 peaked at 14 dpi (Fig. 7B), and the astrocyte number in cluster 1 continuously increased. ST maps characterized the expression of GFAP and Lcn2-the markers for activated astrocyte (Fig. S5D, E).

To further explain the biological processes of the subgroups, we characterized the top 5 DEGs for each cluster (Fig. 7C), and the enriched GO terms based on highly-expressed genes (Fig. 7D). The top terms showed that cluster 0 cells were highly enriched in traditional functions [104], such as neurotransmitter transport and synaptic vesicle cycle. In addition, cluster 0 cells were also significantly enriched for myelination and axonogenesis. The GSVA scores of synaptic vesicle cycle and axon guidance pathway analysis and axon guidance also supported this view (Fig. 7E). Cluster 1 cells were also significantly enriched for ATP metabolic process. The corresponding KEGG analysis showed that cluster 1 cells were highly enriched in cardiac muscle contraction pathway, and the GSVA score showed that it peaked at 7 dpi around the microglial scar (Fig. 7E). The top terms showed that cluster 2 cells were highly enriched in regulate brain energy metabolism and homeostasis [3], such as ATP metabolic process and generation of precursor metabolites and energy, consistent with the previous concepts [20]. Interestingly, cluster 4 cells were significantly enriched for osteoblast differentiation, regulation of ossification, cellular response to Zn^2+^, and cellular response to Cu^2+^. Astrocytes are known to remove excessive K^+^ from the microenvironment [105]. Here, we found that cluster 4 cells might be involved in the homeostasis of Zn^2+^ and Cu^2+^, which cause neuronal cell death [106].

We next applied trajectory analysis (Fig. 7F) and the pseudo-time trajectory algorithm (Fig. 7G). The trajectory direction started from cluster 2 cells to a separate branch consisting of clusters 0, 1, 3, 4, and 5. We found that the genes with significantly elevated levels of expression were associated with the immune response lectin-induced complement pathway, such as C1qa, C1qb, and C1qc (Fig. 7H). Solute carrier family 25 member 22 (Slc25a22), Slc family 7 member 10 (Slc7a10), and Slc family 1 member 2 (Slc1a2) were involved in homeostasis of the excitatory neurotransmitter glutamate, and markedly decreased. At the same time, Slc family 32 member 1 (Slc32a1), involved in gamma-aminobutyric acid (GABA) and glycine uptake into synaptic vesicles, also markedly declined. The relationship between the local excitatory disorder after SCI and the downregulation of these transporters remain unknown [48]. In addition, the pro-regeneration molecular inhibin subunit beta A (Inhba) markedly declined followed by an increase of semaphorin 3B (Sema3b) (Fig. 7I), which inhibits axonal extension. These changes suggested that the microenvironment of the astrocyte scar might switch to inhibit regeneration with the maturation of the scar (Fig. S5F) [67, 107].

Subsequently, we investigated the communication between astrocytes and microglia. The top enriched gene-pairs included Tnfrsf1b_Grn, Tnfrsf1a_Grn, Spp1_Ptger4, Spp1_CD44, Spp1_a9b1 complex, Spp1_a4b1 complex, Plxnb2_Sema4d, Plxnb2_Ptn, Gpr37l1_Pasp, Gpr37_Pasp, CD74_Mif, and CD74_App (Fig. 7J). Interestingly, GPR37L1_PSAP, GPR37_PSAP and PLXNB2_SEMA4D were in the top relationship between microglia and fibroblasts. Here, these pairs were still critical. For example, Gpr37l1_Pasp and Gpr37_Pasp were the top 2 enriched gene-pairs between astrocyte clusters 2/4 and microglia clusters 0/1. In addition, the expression level of Sema4d gradually increased, and the Plxnb2_Sema4d pair [20] between astrocyte clusters 2/4 and microglia cluster 1 were enriched. These data indicated there might be some common mechanisms that mediate cell connections in the scar or these spots represent a mixture of astrocytes and microglia.

Finally, we profiled the single-cell regulatory network inference and clustering (SCENIC) analysis [108] and found the 6 subgroups fell into two major groups (Fig. 7K). The number of cells in group 1 (clusters 0, 1, and 4) increased gradually; groups 1 cells might represent traditional A1 astrocytes [62]. In addition, ST maps showed that group 1 cells wrapped the microglial scar in the intermediate stage (14 and 28 dpi). Interestingly, we found 10 transcription factors that might derive astrocyte transformation to the A1 like-groups 1 cells. In addition, Eph/ephrin signaling can activate the astrocytes which maintain the homeostasis of extracellular glutamate. EphA4 signaling prevents glutamate excitotoxicity [20, 109]. ST maps showed that the expression level of EphA4 was quite low, suggesting a possible neurotransmitter disorder in scar (Fig. S5G). Transglutaminase 2 (TG2) plays a key role in regulating the response of astrocytes to damage. Astrocyte-specific TG2 deletion or inhibition results in a significant improvement in functional recovery after SCI [110]. ST maps showed that TG2 was upregulated in astrocytes in the scar at different time points after SCI (Fig. S5H). Glial BAI3 and BAI1 binding to RTN4/NoGo receptors regulates axonal elongation and synapse formation, thereby controlling neural network activity [111]. Our ST showed that the expression level of Bai3 in the astrocyte scar was low at different time points after SCI (Fig. S5I). In addition, astrocyte-derived saturated lipids contained in apolipoprotein E (APOE) and APOJ lipoparticles mediate toxicity. Eliminating the formation of long-chain saturated lipids by astrocyte-specific knockout of the saturated lipid synthesis enzyme ELOVL1 (elongation of very long chain fatty acid 1) mitigates the astrocyte-mediated toxicity [112]. Our ST showed that activated astrocytes in the scar have high expression of Apoe, Apoj, and Elovl1 (Fig. S5J). An unexpected finding was that activated astrocytes in the scar have high expression of Clu (Fig. S5K), an anti-neuroinflammatory gene. A previous study showed that CLU (Clusterin) is secreted by Schwann cells in peripheral sciatic nerve and induces the outgrowth of sensory neurons but not motor neurons [57]. Recently, an exciting result showed 'runner plasma', collected from voluntarily running mice and infused into sedentary mice, reduces baseline neuroinflammatory gene expression and experimentally-induced brain inflammation. Mechanically, the complement cascade inhibitor CLU in plasma reduces neuroinflammatory gene expression in a mouse model of acute brain inflammation and a mouse model of Alzheimer's disease [113]. The exact role of Clu in SCI remains unknown. In brief, these findings describe the possible roles of astrocytes infiltrating into the scar.

### ST identifies the different oligodendrocyte subtypes in the scar

**Fig. 8:**
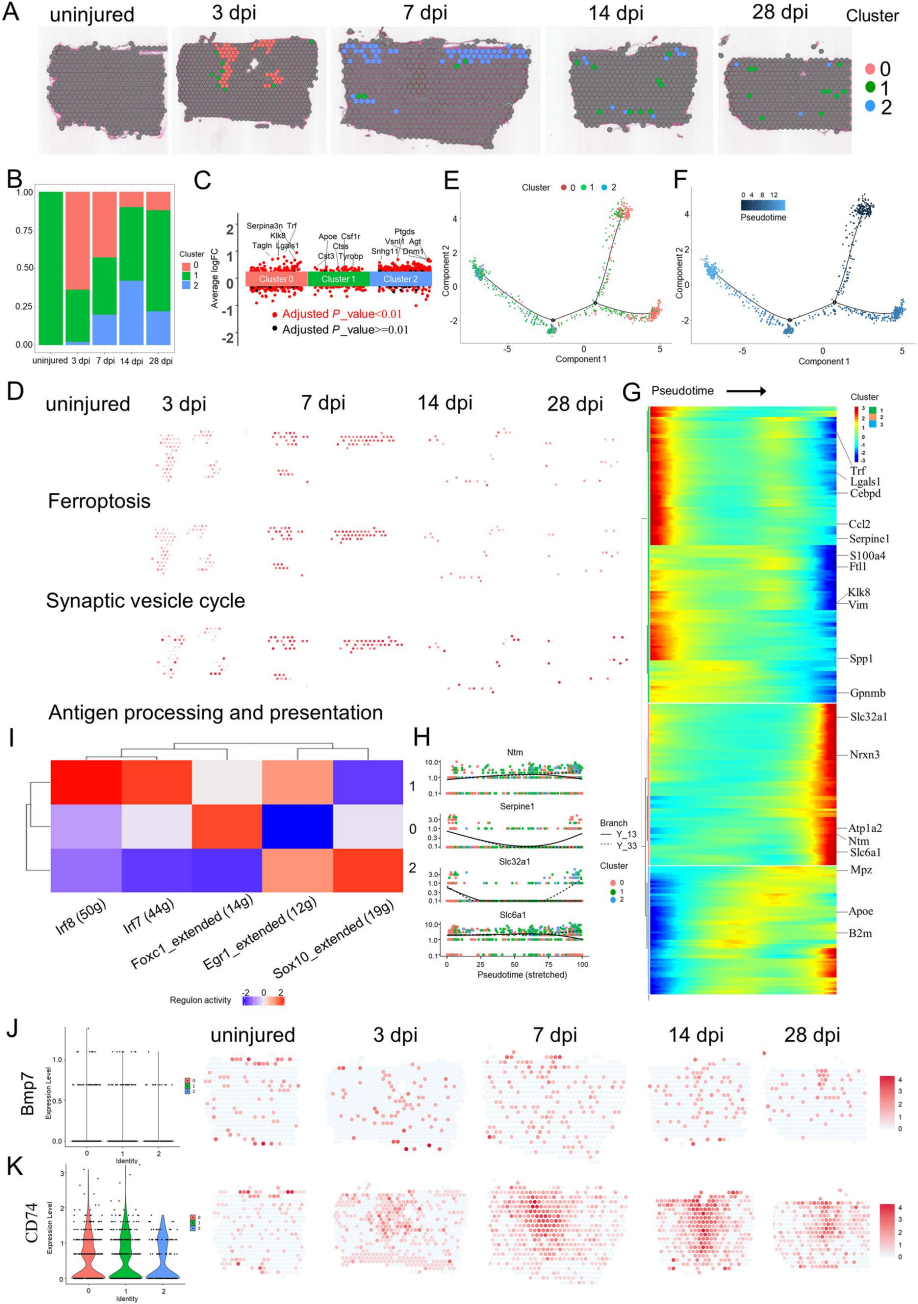
Phenotypic and functional heterogeneity of oligodendrocytes in the glial scar. **A** Spatial maps showing the distribution of 3 oligodendrocyte clusters in the glial scar at different time points after injury. **B** Bar plot showing the fraction of all spots comprising each subpopulation at different time points after injury. **C** Jitterplot showing the top 5 differentially expressed genes in each subpopulation. **D** Spatial maps showing the GSVA scores of ferroptosis pathway enrichment in cluster 0 cells, antigen processing and presentation pathway enrichment in cluster 1 cells, and synaptic vesicle cycle pathway enrichment in cluster 2 cells at different time points after injury. **E**, **F** Trajectory of oligodendrocytes in the glial scar. **G** Heat map showing the changes in gene expression along a spatial trajectory. **H** Expression of Ntm, Serpine1, Slc32a1, and Slc6a1 is correlated with pseudotime. **I** Heat map showing the enriched transcription factors by single-cell regulatory network inference and clustering analysis in different clusters after injury. **J** Violin plots and spatial maps showing the expression of Bmp7 at different time points after injury. **K** Violin plots and spatial maps showing the expression of CD74 at different time points after injury.

Oligodendrocytes wrap around the axons of neurons in the CNS, forming myelin insulators on the surface of neurons that allow electrical signals to travel more efficiently. Interestingly, subpopulations of oligodendrocytes and their progenitor cells have much in common with immune cells in mouse models of multiple sclerosis, and they can be involved in clearing myelin sheaths damaged by disease, in a manner similar to immune cells [114]. We next assessed the cellular heterogeneity among oligodendrocytes in the scar, using re-clustering analysis and visualization on spatiotemporal maps (Fig. 8A) and a UMAP (Fig. S6A, B). We identified three oligodendrocyte subtypes, labeled clusters 0 to 2 (Figs 8B, S6C). Based on the spatiotemporal maps, we found cluster 0 cells were mainly distributed in the outer layer of macrophages in the scar core at 3 dpi and diminished rapidly. By contrast, cluster 2 cells were mainly distributed in the white matter of the scar (Fig. 8A). ST maps also characterized the expression of Mbp, Mog, Mag, and Cldn11 – markers for activated oligodendrocytes (Fig. S6D, E).

Subsequently, we characterized the top 5 DEGs in each cluster (Fig. 8C), and the enriched GO terms based on highly-expressed genes (Fig. S6F). The top terms showed that cluster 0 cells were highly enriched in response to wounding and epithelial cell proliferation. The GSVA score of ferroptosis signaling pathway analysis also supported this view (Fig. 8D). But cluster 0 cells diminished gradually. In addition, cluster 1 cells were significantly enriched in neutrophil activation involved in immune response, leukocyte-mediated immunity, antigen processing and presentation, and macrophage activation. The GSVA score of antigen processing and presentation analysis enriched in cluster 1 cells significantly increased from 7 dpi to 28 dpi (Fig. 8D), suggesting that they work in a manner similar to immune cells [114]. Cluster 2 cells were significantly enriched for pre-synapse. The GSVA score of synaptic vesicle cycle enriched in cluster 2 cells was significantly increased at 7 dpi (Fig. 8D).

The trajectory analysis (Fig. 8E) and the pseudo-time trajectory algorithm (Fig. 8F) were then applied, and we found that the expression level of serpin family E member 1 (Serpine1), a component of innate immunity, gradually declined. But beta-2-microglobulin, a component of the class I major histocompatibility complex (MHC) involved in the presentation of peptide antigens to the immune system, gradually increased (Fig. 8G). Galectin 1, which plays a role in regulating apoptosis, gradually declined. Interesting, Slc family 6 member 1 (Slc6a1) and Slc family 32 member 1 (Slc32a1) mediate the rapid removal of GABA and maintain low extracellular levels. Their expression levels significantly increased, suggesting that oligodendrocytes participate in the regulation of excitability in the scar. In addition, the cell adhesion molecule neurotrimin (Ntm), which has an inhibitory impact on neurite outgrowth [115], showed a significantly increased expression level during the maturation of the scar (Fig. 8H).

SCENIC analysis found that Foxc1 potentially controlled cluster 0 cells (Fig. 8I). Foxc1 is an important regulator of cell viability and resistance to oxidative stress in the eye [116]. Irf7 and Ifr8 might be involved in the cell fate of cluster 1 cells. Interesting, Sox10 controlled the cell fate of cluster 2 cells. Sox10 has been confirmed to play a central role in oligodendrocyte maturation and CNS myelination. Specifically, Sox10 activates the expression of myelin genes such as Dusp15 and Myrf during oligodendrocyte maturation [117].

Oligodendrocyte death after SCI contributes to the demyelination of spared axons. Bone morphogenic protein-7 (BMP7) potently inhibits TNF-α-induced oligodendrocyte apoptosis [118]. Overexpression of BMP7 reduces oligodendrocyte loss and promotes functional recovery after SCI [119]. In our data, ST maps showed that cells secreting BMP7 were mainly in the fibroblast scar, suggesting a potential beneficial role (Fig. 8J). Last, ST maps characterized the expression of the CD74-a component of class II MHC (Fig. 8K). CD74 is a critical chaperone that regulates antigen presentation for the immune response [120]. All three oligodendrocyte subtypes had a moderate expression of CD74. Taken together, these findings deciphered the potential functions of oligodendrocytes infiltrating into the scar.

### Gene Regulatory Networks Controlling Scar Programming

**Fig. 9:**
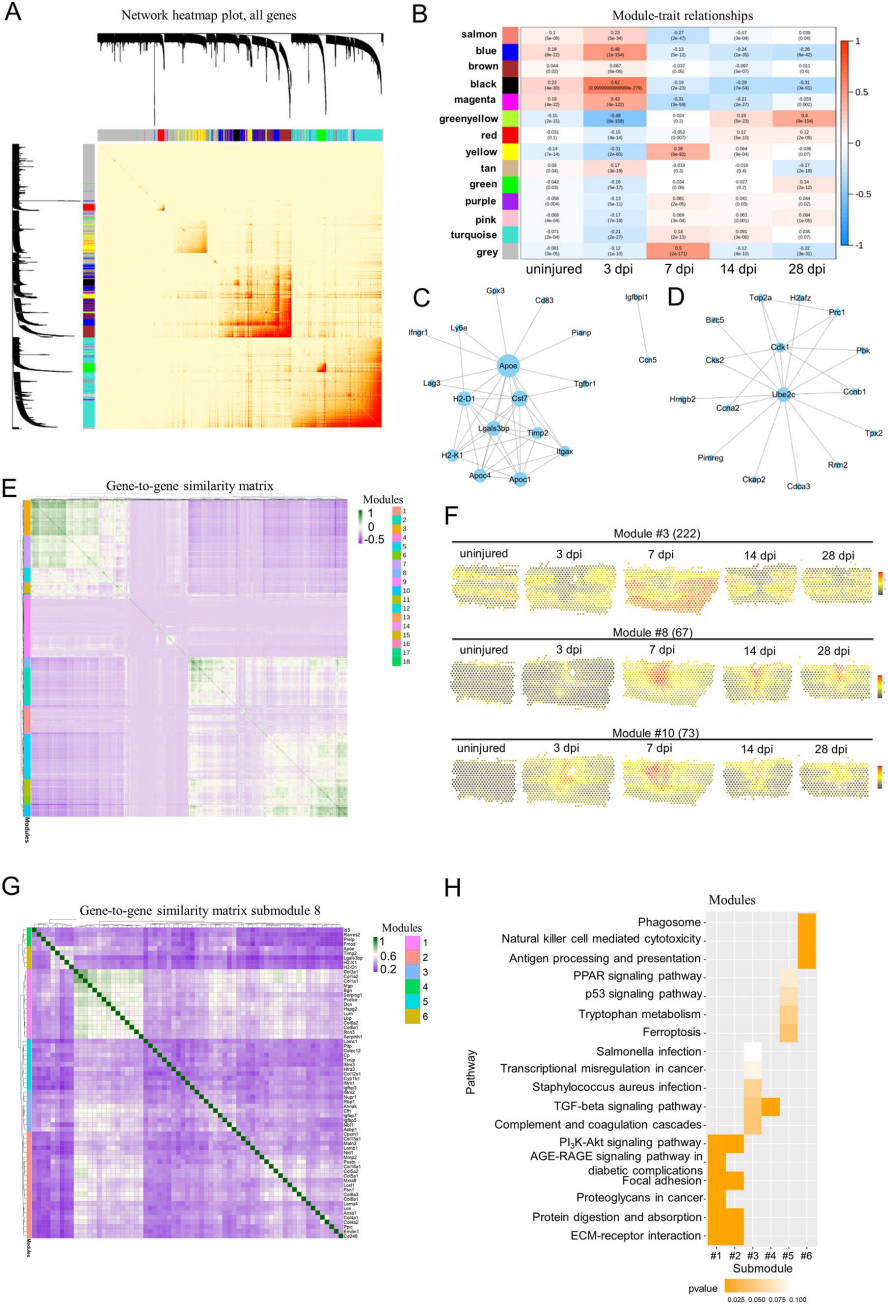
Spatiotemporal dynamics of gene expression during maturation of the scar. **A** Hierarchical clustering showing the network of the correlation of module membership and gene significance, as well as the correlation among all genes. **B** Heatmap depicting the correlation scores (digit in the box above) as well as its corresponding P-value (digit in the box below) of modules (rows) and different times after injury (columns). **C** Co-expression of selected hub gene enrichment in Megreenyellow. The networks are created by Cytoscape. **D** Co-expression network of selected hub gene enrichment in Meblue using Cytoscape. **E** Biclustering of the spatial gene expression measurements reveal spatially and temporally co-expressed genes. Identifiers of co-expression modules are listed. **F** Average spatiotemporal expression dynamics of modules 3, 8, and 10. **G** Hierarchical clustering of genes in module 8. **H** Analysis of enriched KEGG pathways among the genes for the submodules in **G**.

To better understand scar-relevant changes in gene regulation and interactions between cell types, we carried out WGCNA and an unbiased co-expression analysis [37] of our ST data. In WGCNA, 2,000 genes (Table S4) from the spatiotemporal transcriptome dataset in clusters 2 and 3 (Fig. 1H) were re-clustered. The branches of highly-correlating genes were formed, which were cut and assigned a color. Finally, the correlation of genes and modules was calculated (Fig. 9A) and 14 modules were identified (Figs 9B, S7A). Among the 14 modules, the green-yellow module had the most significant correlation with time (Fig. S7A); this gradually increased following scar formation and approximately represented microglia and astrocyte (Fig. 9B). We next used the network of the enriched hub module genes involved in green-yellow to identify key controlling genes (Fig. 9C). The blue and black modules had the most significant correlations with the early acute stage (3 dpi) and might represent macrophages and oligodendrocytes, and hub module genes in blue (Fig. 9D) and in black (Fig. S7B) were each applied out.

Subsequently, we adapted another unbiased co-expression analysis more suitable for ST data [37]. We identified 18 major co-expression modules on the basis of 2,000 highly-variable genes from the spatiotemporal transcriptome dataset (Table S5). Among them, modules 3, 8, 10, 16, and 17 were highly correlated and used as candidate analysis modules (Fig. 9E). ST maps showed the spatiotemporal activity of modules 3, 8, and 10 (Fig. 9F). We next grouped the genes of modules 3, 8, and 10 on the basis of their expression pattern, resulting in submodules (Figs 9G, S7C–F). Col3a1, col1a2, and col1a1 (submodule 8.1) had the highest correlation score (Fig. 9G). KEEG pathway enrichment analysis of the submodule 8.1 showed that it was enriched for ECM–receptor interaction (Fig. 9H). Gpnmb, Lyz2, Ftf1, Lgals3, Ctsd, and Ctsb (submodule 10.3) had the highest correlation score (Fig. S7E). KEEG pathway enrichment analysis of submodule 10.3 showed that it was enriched for lysosomes (Fig. S7F). Collectively, the pathway activity encompassed by modules 8 and 10 might reveal signaling between cell types during glial and fibroblastic activation in the scar.

#### The Scar Boundary is Interpreted by ST

**Fig. 10:**
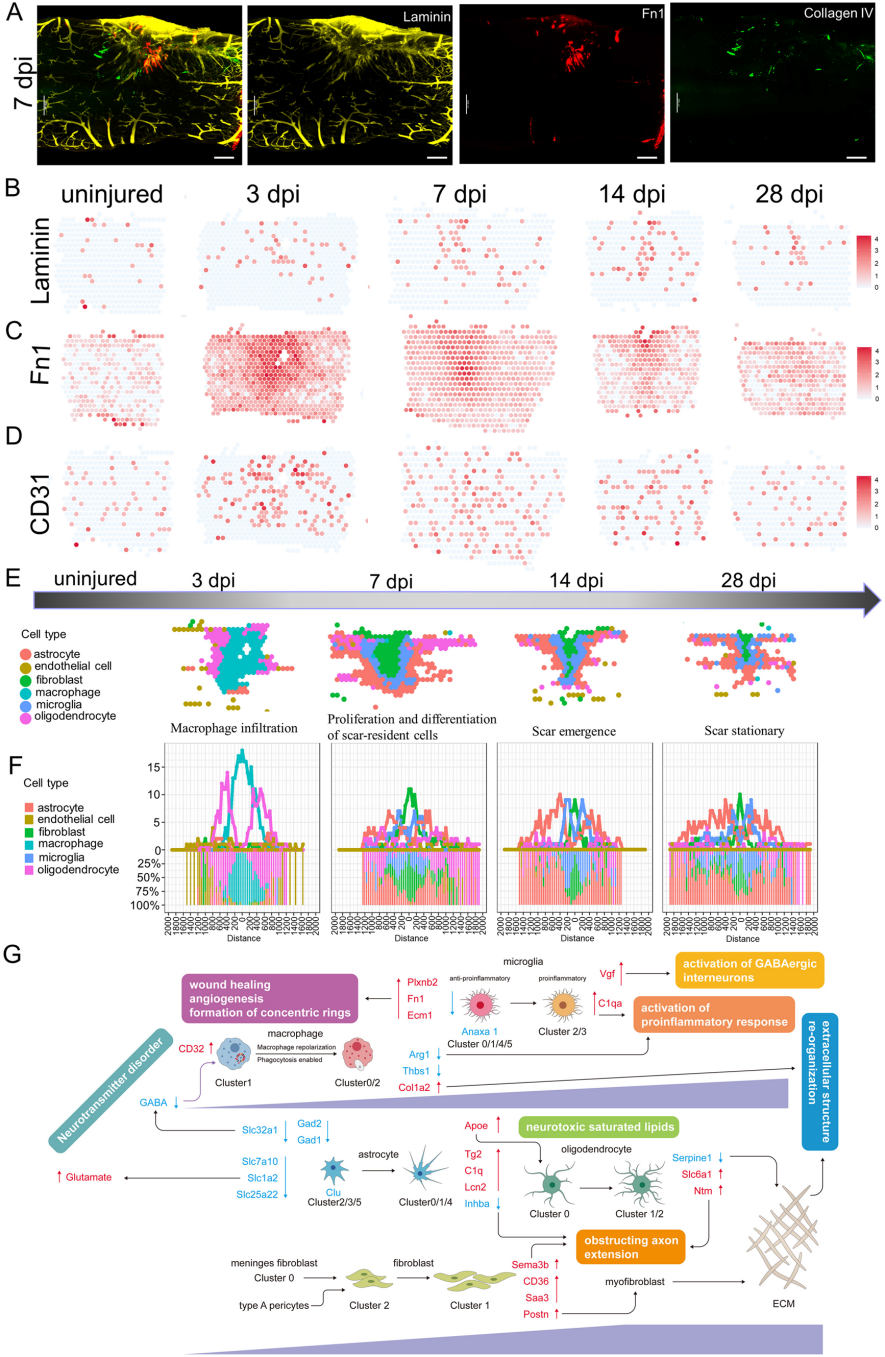
Visualization and interpretation of the scar with transparency and ST. **A** IF-stained 7-dpi scars for laminin (yellow), Fn1 (red), and collagen IV (green) showing the extent of the scar boundary (scale bars, 200 µm). **B–D** Spatial maps showing the expression of laminin (**B**) Fn1 (**C**), and CD31 (**D**) at different time points after injury. **E** ST showing the extent of the scar boundary of different cell types. **F** Quantification of the number and fraction of spots representing astrocytes, endothelial cells, fibroblasts, macrophages, microglia, and oligodendrocytes. **G** Summary of the changes in key genes and biological processes after SCI.

The traditional characteristics of THE lesion site in mice after adult injury lack of structure visualization with immunofluorescence for laminin, fibronectin, chondroitin sulfate proteoglycan, GFAP, CD68, CD31, and collagen [10, 20, 93]. All these data were derived from sections and we lack a global understanding of its distribution throughout the spinal cord scar. Although diffusion tensor imaging can provide accurate information on the cavity area after SCI, it is difficult to distinguish cell types by this method [121, 122]. With the help of tissue transparency, laminin, fibronectin, and collagen were re-characterized in our mouse model of T10 right lateral hemisection (Fig. 10A). Laminin was highly enriched in the wound area at 7 dpi. On the contrary, fibronectin and collagen had relatively low expression, different from the expression pattern in neonatal mice with spinal cord crush [93]. We then analyzed our ST data for laminin (Fig. 10B) and fibronectin (Fig. 10C). Overall, the ST maps showed that fibronectin was upregulated in the injury site and roughly characterized the region of the scar at 3, 7, and 14 dpi. Furthermore, the ST maps showed that laminin was upregulated in the injury site and clearly characterized the region of the scar at 7, 14, and 28 dpi. In addition, the ST maps of CD31 showed that neovascularization failed to penetrate the scar (Fig. 10D), similar to stained slices [93]. Collectively, combining the ST maps of scar markers to describe the size of the scar is a potential breakthrough. To describe the distribution of each cell type more accurately, we counted the number and calculated the fraction of each cell type maintaining scar architecture at different times after SCI (Fig. 10E, F), and found that fibroblasts were always in the center during scar shrinkage and maturation. Our findings have clearly confirmed the previous concept that a fibrotic scar is surrounded by a microglial scar, which is wrapped by an astrocytic scar [3, 10, 13, 20]. Furthermore, microglial scars and astrocytic scars in the gray matter did shrink, but we found that they expanded to a larger area along the white matter at 28 dpi (Fig. 10E, F). A recent study also confirmed the diffusion of the wound response to adjacent and remote areas [123]. Conventional wisdom holds that secondary injury following SCI can be temporally divided into acute, sub-acute, and chronic phases [1, 3, 10, 13, 20, 65]. After combining the possible cell type, functional diversity, trajectory, and the fraction of scar-resident cells, we propose four possible phases of scar formation: macrophage infiltration, proliferation and differentiation of scar-resident cells, scar emergence, and stationary scar. Taken together, we describe the spatiotemporal dynamics of key genes in specific cell types, thus providing a global atlas of the gene expression and molecular regulation driving scar formation following SCI (Fig. 10G).

## Discussion

Following injury to the CNS, scar formation is essential to seal the damaged tissue and control the expansion of damage, but it is also a physical barrier to regeneration. The spinal cord scar is known to be a compartmentalized structure that contains a variety of cells. For example, activated astrocytes surround the outer layer of fibrous scar and constitute the glial component of the scar. Non-neural cells, including local and infiltrating immune cells and fibroblasts that produce extracellular matrix, gather in the core of the scar, and constitute the fibrotic or matrix component of the scar [10]. Although many different types of cell are thought to be involved in scars, it is difficult to accurately track and assess their genetic fate *in vivo* due to the lack of lineage-tracing systems for all the corresponding cells. scRNA-seq allows the study of cellular heterogeneity in the scar [20, 21]. However, scRNA-seq generally does not show spatial patterns of gene expression. Here, for the first time, we measured gene expression in the mouse spinal cord during scar formation using ST and described the spatial heterogeneity that single-cell technology cannot achieve, providing a resource map of scar formation after spinal hemisection.

### Verification of Scar Components and Phase Division Obtained using the ST Technique

Conventional wisdom describes the mature CNS scar as a compartmentalized and multicellular structure [10, 13, 77]. Using ST technology, we found that activated oligodendrocytes surrounded the outer layer and might be involved in immune homeostasis. Scar-resident oligodendrocytes also obstruct axon extension through secreted Ntm. In addition, the traditional view argues that scar-resident macrophages come from the blood supply [68, 74]. But recent studies have shown that monocytes can come from adjacent skull and spine bone marrow after SCI [76]. Our ST diagrams also revealed that the majority of scar-resident macrophages were derived from adjacent skull in our model. The pathophysiology of SCI involves primary and secondary mechanisms of injury. The concept of secondary SCI was first introduced by Allen [124].; it begins within minutes following the initial primary injury and continues for weeks or months, eventually causing the formation of a scar surrounding the lesion site [125]. To describe the pathophysiological characteristics of the spinal cord tissue surrounding the lesion site, a division of scar formation into phases was proposed. Specially, the acute phase (˂7 dpi) begins immediately following SCI [125] and the sub-acute phase begins at 7–14 dpi, involving the demyelination of surviving axons, axonal dieback, and matrix remodeling [1]. Further changes occur in the chronic phase (˃14 dpi), which includes progressive axonal die-back and maturation of the glial scar [126]. However, some researchers have proposed an intermediate phase (14 dpi–6 months later) and a chronic phase (˃6 months later) [66]. To classify the cellular, molecular, and biochemical phenomena that continued as the spinal cord tissue self-destructed and impeded neurological recovery in the model with T10 right lateral hemisection, we propose four possible phases of scar formation: macrophage infiltration, proliferation and differentiation of scar-resident cells, scar emergence, and stationary scar.

### Spatiotemporal-specific Action of the Glial Scar in Spinal Cord Repair Deciphered by the ST Technique

The current general view of glial scars is that they have detrimental properties (inhibiting axon regeneration) and beneficial properties (protecting spared neural tissue), but their exact time-specific roles are still poorly understood [24, 81]. Although scar-resident fibroblasts obstructed axon extension at the intermediate stage (14 and 28 dpi), they also secreted Bmp7, which might support the survival of scar-resident oligodendrocytes. During the processes of gliosis and fibrosis, the bidirectional ligand-receptor interactions on the cluster boundary help to maintain the scar structure. The spatiotemporal changes of a cell's position in the scar may be related to its function. A special subset of neonatal microglia can form bridges between the two stumps after SCI and mediate axon regeneration [93]. Through ST analysis, we also found that a special subset of adult microglia can cross the fibrotic scar and build a bridge. But these bridges eventually disappeared at 28 dpi, suggesting that differences in regenerative ability at different stages of development are closely related to the scar microenvironment. ST also determined that Cluster 1 cells were likely to be M2 macrophages and always in the core of the injury site. Interesting, Cluster 1 cells had a high expression level of Ccr2, suggesting that they might be bone marrow-derived mononuclear macrophages.

### Treatment Strategies to Eliminate Scars Provided by ST Technology

In recent years, the reconstruction of the regenerative microenvironment based on biomaterials and the promotion of functional rehabilitation have become one of the frontiers of SCI treatment. The ideal scaffolds have good biocompatibility, biodegradability, and biomechanical properties. These characteristics help to more effectively repair an SCI through reconstruction of the regenerative microenvironment [127]. For example, neurotrophin-3-loaded chitosan biodegradable material [128] and small molecules combined with collagen hydrogel [129] have enabled robust neural regeneration and functional restoration. To reduce microglia/macrophage-mediated inflammation, a combination of photo-crosslinked hydrogel transplantation and colony-stimulating factor 1 receptor (CSF1R) inhibitor PLX3397 treatment inhibits pro-inflammatory factors *via* the depletion of activated microglia/macrophages and promotes the neurogenesis of endogenous neural stem/progenitor cells [130]. Spatial transcriptomics technology has provided a spatial composition map and has gradually become a powerful tool to decipher tissue development and pathology [131]. Apart from microglia/macrophages, our ST found that both astrocytes and oligodendrocytes might be involved in maintaining inflammatory homeostasis. Specifically, the effects of oligodendrocytes are not well understood. In addition, CLU derived from plasma has been demonstrated to be a pivotal anti-inflammatory effector in mouse models of acute brain inflammation and Alzheimer's disease [113]. The CLU-rs9331896-TT genotype is even a risk factor for Parkinson's disease [132, 133]. ST showed that activated astrocytes had a high expression level of Clu in the acute and subacute stages, and gradually decreased in the intermediate stage. CLU-loaded biodegradable material can control the release rate and might help to reverse the deficiency of CLU in the intermediate stage for the treatment of glial scars.

Another issue is the persistence of CD36^+^ myofibroblasts in the scar core. Tissue-resident fibroblasts are known to play a role in scar fibrosis [134, 135], and have emerged as a key cell type in regulating the activation or suppression of the immune response [136], For example, dermal fibroblasts that recruit and activate CD8^+^ cytotoxic T cells through secreted chemokines are responsible for driving patterned autoimmune activity in vitiligo [137]. In our model, SCI induced a significant fibroblast response, which formed a fibrotic scar that restricts the regeneration of adult mammalian axons. Both our ST and previous studies have established that scar-associated fibroblasts are derived from type A pericytes [138]. We hypothesize that functional recovery might be promoted by eliminating scar-associated fibroblasts. Fortunately, a moderate reduction in pericyte-derived fibrosis using a genetic strategy can reduce scar pathology and achieve functional recovery [83]. ST analysis showed that CD36, which is involved in skin scar formation, was markedly activated in scar-resident fibroblasts. This suggests that CD36 may be a therapeutic target for spinal cord scars. Salvianolic acid, an inhibitor of CD36, may become a new therapy to eliminate scar-resident fibroblasts [45, 139, 140]. After treating mice with SAB, we indeed found that the fibrosis was reduced. In addition, during skin wound healing in the mouse, adipocytes regenerate from myofibroblasts, a cell type thought to be differentiated and non-adipogenic [141], suggesting the possibility that scar-resident fibroblasts can be eliminated by reprogramming [142].

Spatial transcriptomics maps of healthy or diseased tissues facilitate unbiased exploration and hypothesis generation. We found that the spatial mapping of the differentiation transition more comprehensively described the possible trajectories between glial cells, fibroblasts, and immune cell subpopulations, and more accurately defined the scar boundary and the extent of the lesion. Thus far, the current ST method still faces challenges, including limitations of resolution and sensitivity as well as throughput. But, with the continuous updating of technology, these problems are being gradually overcome [131]. Here, we used the spatial mapping maps of known typical markers in different cell types to verify the spot division after applying the AddModule Score function from the Seurat R package. Fortunately, we found that each cluster was the most likely reasonable cell type shown in the previous studies [10, 20, 21].

Our transcriptome data set aimed to present a decoding of glial scars, and to serve as a reference for future research. We believe that our data will help understand the spatial gene expression and gene regulatory network of spinal glial scars, and provide ideas for potential clinical treatment strategies. Last, we present all aspects of these highly dimensional data *via* the spatio-temporal cell atlas of the mouse spinal cord scar (STASCS) (http://47.92.172.28:12200).

## Supplementary Information

Below is the link to the electronic supplementary material.

Supplementary file 1 (PDF 2824 KB)

Supplementary file 2 (XLSX 1382 KB)

Supplementary file 3 (XLSX 9 KB)

Supplementary file 4 (XLSX 825 KB)

Supplementary file 5 (XLSX 30 KB)

Supplementary file 6 (XLSX 35 KB)
