# Supplementary material for: Spatiotemporal Dynamics of the Molecular Expression Pattern and Intercellular Interactions in the Glial Scar Response to Spinal Cord Injury
Source: Neurosci Bull. 2022 Jul 5;39(2):213–44. doi: 10.1007/s12264-022-00897-8 (PMC9905408; doi:10.1007/s12264-022-00897-8)
Supplement: Supplementary file 1 — (PDF 2824 KB) [file 12264_2022_897_MOESM1_ESM.pdf]

Supplementary Materials

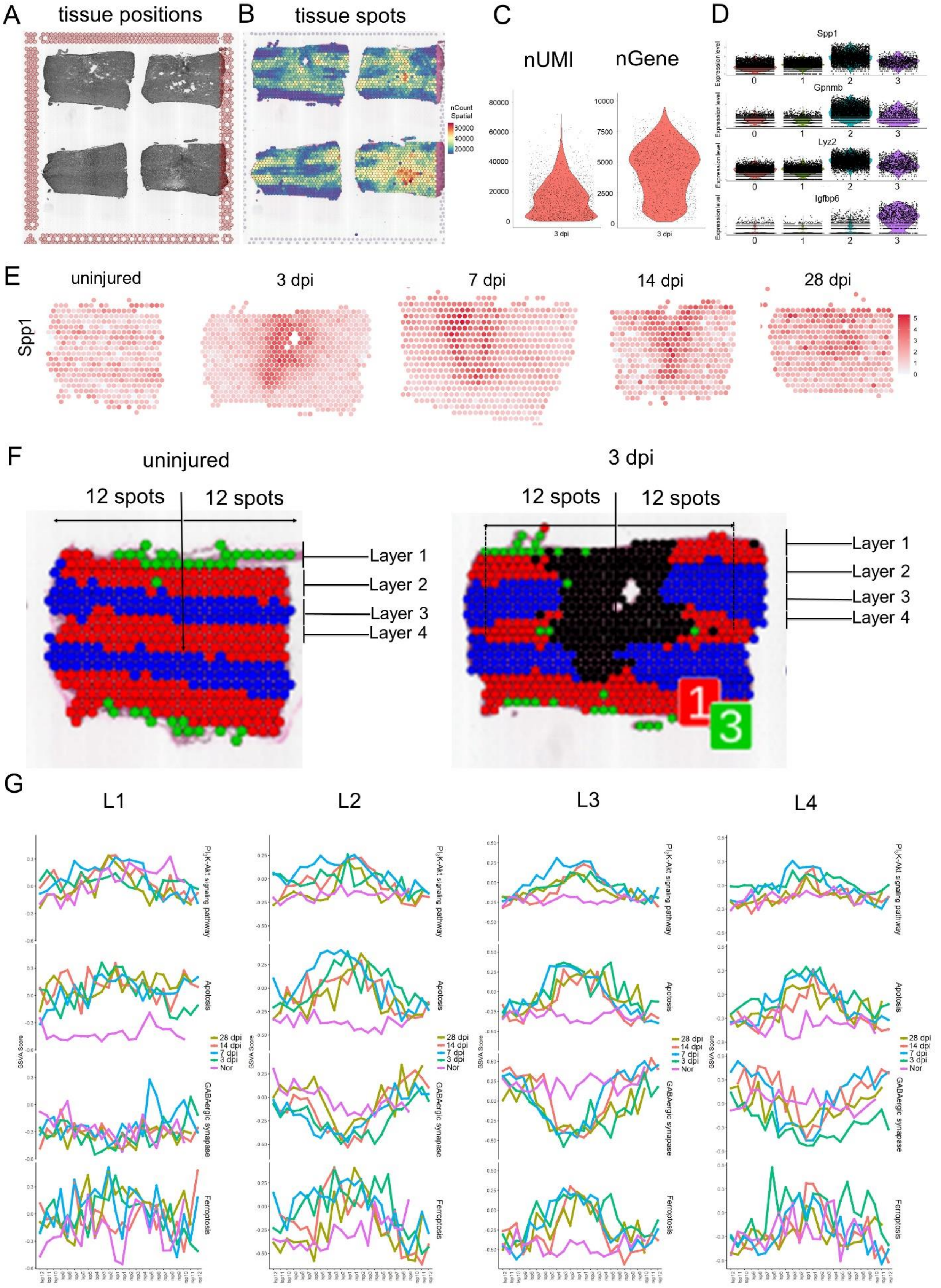

Fig. S1 Quality control and spatial distribution of spots involved in glial scar formation. A Representative images of section positions from samples at 3 dpi. **B** Representative images of the captured spots in sections from samples at 3 dpi. **C** Violin plots showing nUMI and nGene in sections from the samples at 3 dpi. **D** Violin plots showing representative genes of cluster 2 cells and cluster 3 cells, the scar-associated clusters. **E** Spatial feature plots of Spp1, a stress response-associated gene. **F** Symmetrical distribution pattern of cluster 0 cells and cluster 1 cells in normal spinal cord; these cells were divided into four layers from layer 1 to layer 4. Subsequently, these cells in the scar were divided into four layers by similar approaches. **G** Selected Gene Set Variation Analysis terms that were enriched in different layers at different stages of scar maturation. Fisher's exact test was used in the pathway enrichment.

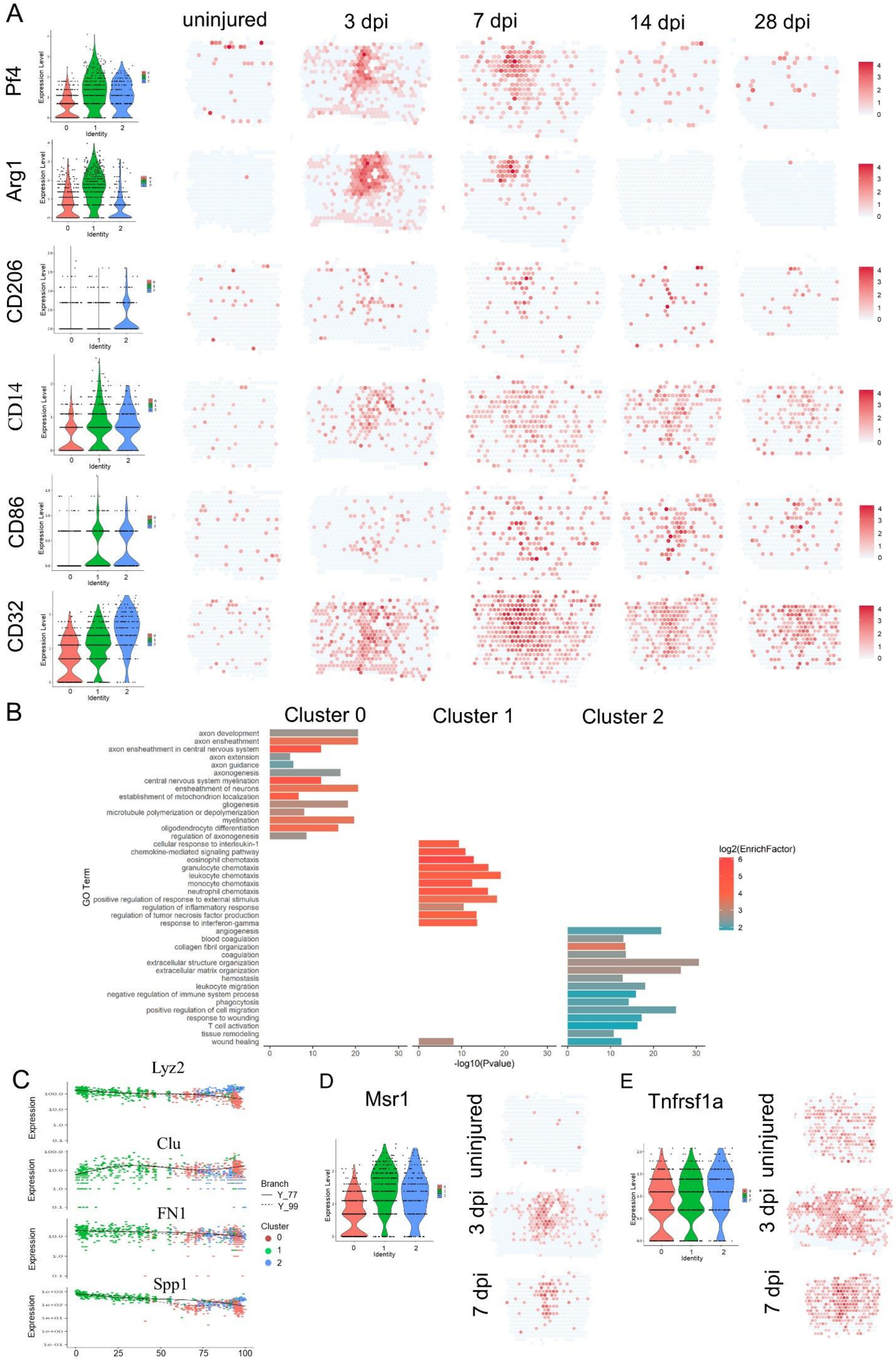
A
uninjured
3 dpi
7 dpi
14 dpi
28 dpi
Pf4
Arg1
CD206
CD14
CD86
CD32
Expression Level
Identity
B
Cluster 0
Cluster 1
Cluster 2
GO Term
axon development
axon ensheathment
axon ensheathment in central nervous system
axon extension
axon guidance
axonogenesis
central nervous system myelination
ensheathment of neurons
establishment of mitochondrion localization
gliogenesis
microtubule polymerization or depolymerization
myelination
oligodendrocyte differentiation
regulation of axonogenesis
cellular response to interleukin-1
chemokine-mediated signaling pathway
eosinophil chemotaxis
granulocyte chemotaxis
leukocyte chemotaxis
monocyte chemotaxis
neutrophil chemotaxis
positive regulation of response to external stimulus
regulation of inflammatory response
regulation of tumor necrosis factor production
response to interferon-gamma
angiogenesis
blood coagulation
collagen fibril organization
coagulation
extracellular structure organization
extracellular matrix organization
hemostasis
leukocyte migration
negative regulation of immune system process
phagocytosis
positive regulation of cell migration
response to wounding
T cell activation
tissue remodeling
wound healing
log2(EnrichFactor)
-log10(Pvalue)
C
Lyz2
Clu
FN1
Spp1
Expression
Branch
Y_77
Y_99
Cluster
D
Msr1
E
Tnfrsf1a

Fig. S2 Distinct macrophage responses after lateral hemisection of the spinal cord. A Violin plots and spatial maps showing the expression of marker genes at different time points after injury. **B** Selected GO terms that were enriched for each cluster (Fisher's exact test). **C** Scatterplots of expression dynamics of selected genes along pseudotime. **D**, **E** Violin plots and spatial maps showing the expression of selected genes at different time points after injury.

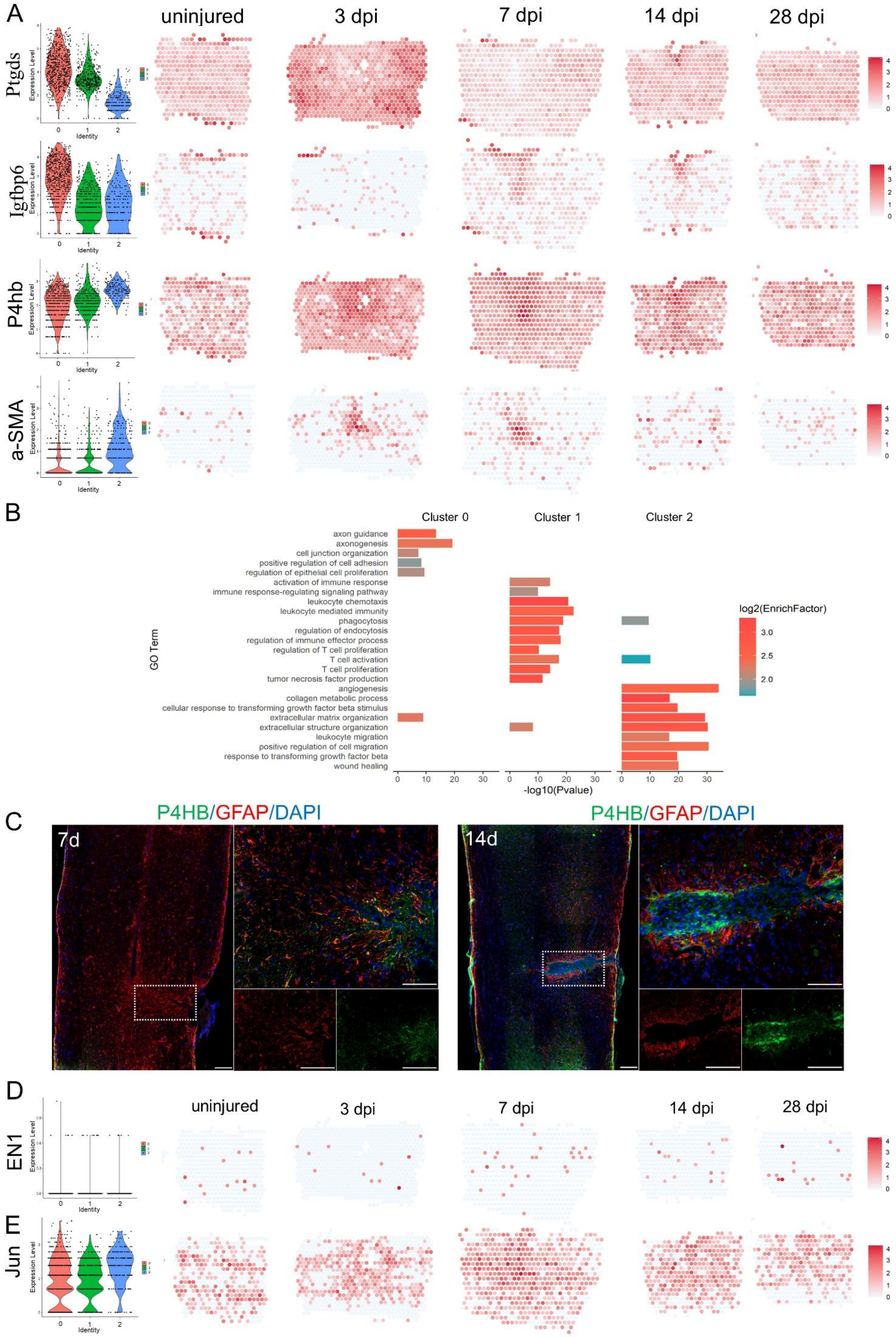
A
uninjured
3 dpi
7 dpi
14 dpi
28 dpi
Ptgds
Igfbp6
P4hb
a-SMA
Expression Level
Identity
B
Cluster 0
Cluster 1
Cluster 2
GO Term
axon guidance
axonogenesis
cell junction organization
positive regulation of cell adhesion
regulation of epithelial cell proliferation
activation of immune response
immune response-regulating signaling pathway
leukocyte chemotaxis
leukocyte mediated immunity
phagocytosis
regulation of endocytosis
regulation of immune effector process
regulation of T cell proliferation
T cell activation
T cell proliferation
tumor necrosis factor production
angiogenesis
collagen metabolic process
cellular response to transforming growth factor beta stimulus
extracellular matrix organization
extracellular structure organization
leukocyte migration
positive regulation of cell migration
response to transforming growth factor beta
wound healing
-log10(Pvalue)
log2(EnrichFactor)
C
P4HB/GFAP/DAPI
7d
14d
D
EN1
E
Jun

Fig. S3 Distinct fibroblast responses after lateral hemisection of the spinal cord. A Violin plots and spatial maps showing the expression of selected differentially-expressed genes and marker genes at different time points after injury. **B** Selected GO terms enriched for each cluster (Fisher's exact test). **C** IF-stained 7- and 14-dpi scars for GFAP (red), P4HB (green), and DAPI (blue) confirming the maturation of the scar (*n* = 3; scale bars, 200 μm). **D** Violin plots and spatial maps showing the expression of En1 and Jun, critical regulators of pathological skin scarring, at different time points after injury.

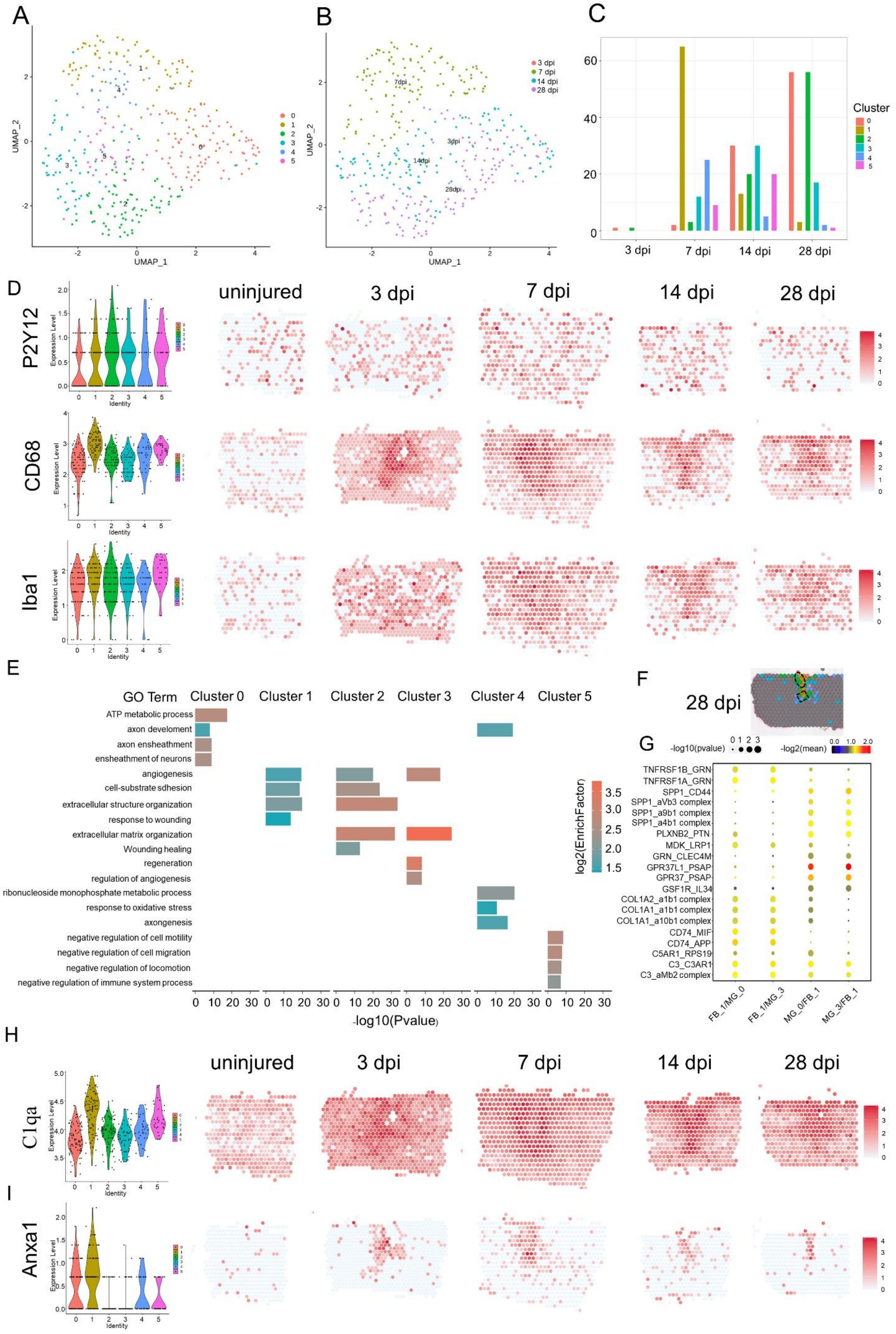
A
B
C
UMAP_1
UMAP_2
3 dpi
7 dpi
14 dpi
28 dpi
Cluster
D
P2Y12
CD68
Iba1
Expression Level
Identity
uninjured
3 dpi
7 dpi
14 dpi
28 dpi
E
GO Term
Cluster 0
Cluster 1
Cluster 2
Cluster 3
Cluster 4
Cluster 5
ATP metabolic process
axon develoment
axon ensheathment
ensheathment of neurons
angiogenesis
cell-substrate sdhesion
extracellular structure organization
response to wounding
extracellular matrix organization
Wounding healing
regeneration
regulation of angiogenesis
ribonucleoside monophosphate metabolic process
response to oxidative stress
axongenesis
negative regulation of cell motility
negative regulation of cell migration
negative regulation of locomotion
negative regulation of immune system process
log2(EnrichFactor)
-log10(Pvalue)
F
28 dpi
G
-log10(pvalue)
-log2(mean)
TNFRSF1B_GRN
TNFRSF1A_GRN
SPP1_CD44
SPP1_aVb3 complex
SPP1_a9b1 complex
SPP1_a4b1 complex
PLXNB2_PTN
MDK_LRP1
GRN_CLEC4M
GPR37L1_PSAP
GPR37_PSAP
GSF1R_IL34
COL1A2_a1b1 complex
COL1A1_a1b1 complex
COL1A1_a10b1 complex
CD74_MIF
CD74_APP
C5AR1_RPS19
C3_C3AR1
C3_aMb2 complex
FB_1/MG_0
FB_1/MG_3
MG_0/FB_1
MG_3/FB_1
H
C1qa
I
Anxa1

Fig. S4 Distinct microglial responses after lateral hemisection of the spinal cord. A UMAP spots showing the 3 clusters of microglia in the glial scar. **B** UMAP spots embedding overlay showing the distribution of spots at different time points after injury. **C** Histogram showing the number of spots in each subpopulation at different time points after injury. **D** Violin plots and spatial maps showing the expression of selected marker genes at different time points after injury. **E** Selected GO terms that were enriched for each cluster (Fisher's exact test). **F** Definition of the boundary areas to study the interaction between two neighboring microglia clusters in the scar at 28 dpi. **G** Dot plot of the mean interaction scores between neighboring clusters at 28 dpi. The size of a circle denotes the p-value, and the color denotes the mean interaction score. **H** Violin plots and spatial maps showing the expression of C1q and Anxa1 at different time points after injury.

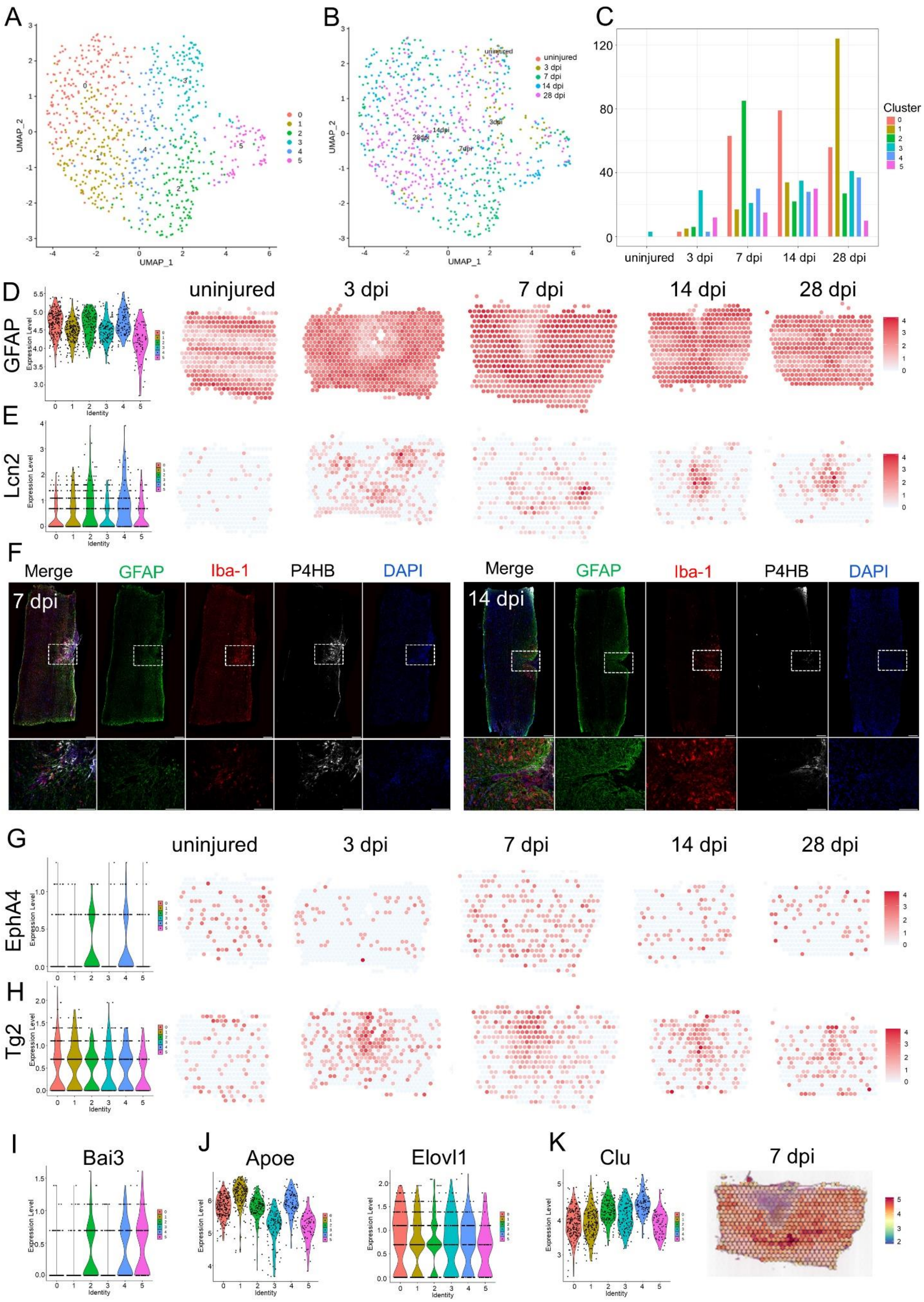

A
B
C
uninjured
3 dpi
7 dpi
14 dpi
28 dpi
Cluster
UMAP_1
UMAP_2
D
GFAP
E
Lcn2
F
Merge
GFAP
Iba-1
P4HB
DAPI
G
EphA4
H
Tg2
I
Bai3
J
Apoe
Elovl1
K
Clu
Expression Level
Identity

Fig. S5 Distinct astrocyte responses after lateral hemisection of the spinal cord

A UMAP spots showing the 6 clusters of astrocytes in the glial scar. **B** UMAP spots embedding overlay showing the distribution of spots at different time points after injury. **C** Histogram showing the number of spots in each subpopulation at different time points after injury. **D, E** Violin plots and spatial maps showing the expression of GFAP (**D**) and Lcn2 (**E**) at different time points after injury. **F** IF-stained 7- and 14-dpi scars for GFAP (green), Iba1(red), P4HB (gray), and DAPI (blue) (*n* = 3, scale bar, 200 μm). **G, H** Violin plots and spatial maps showing the expression of EphA4 (**G**) and Tg2 (**H**) at different time points after injury. **I–K** Violin plots showing the expression of Bai3 (**I**), Apoe and Elovl1 (**J**) and Clu in the astrocyte scar after injury.

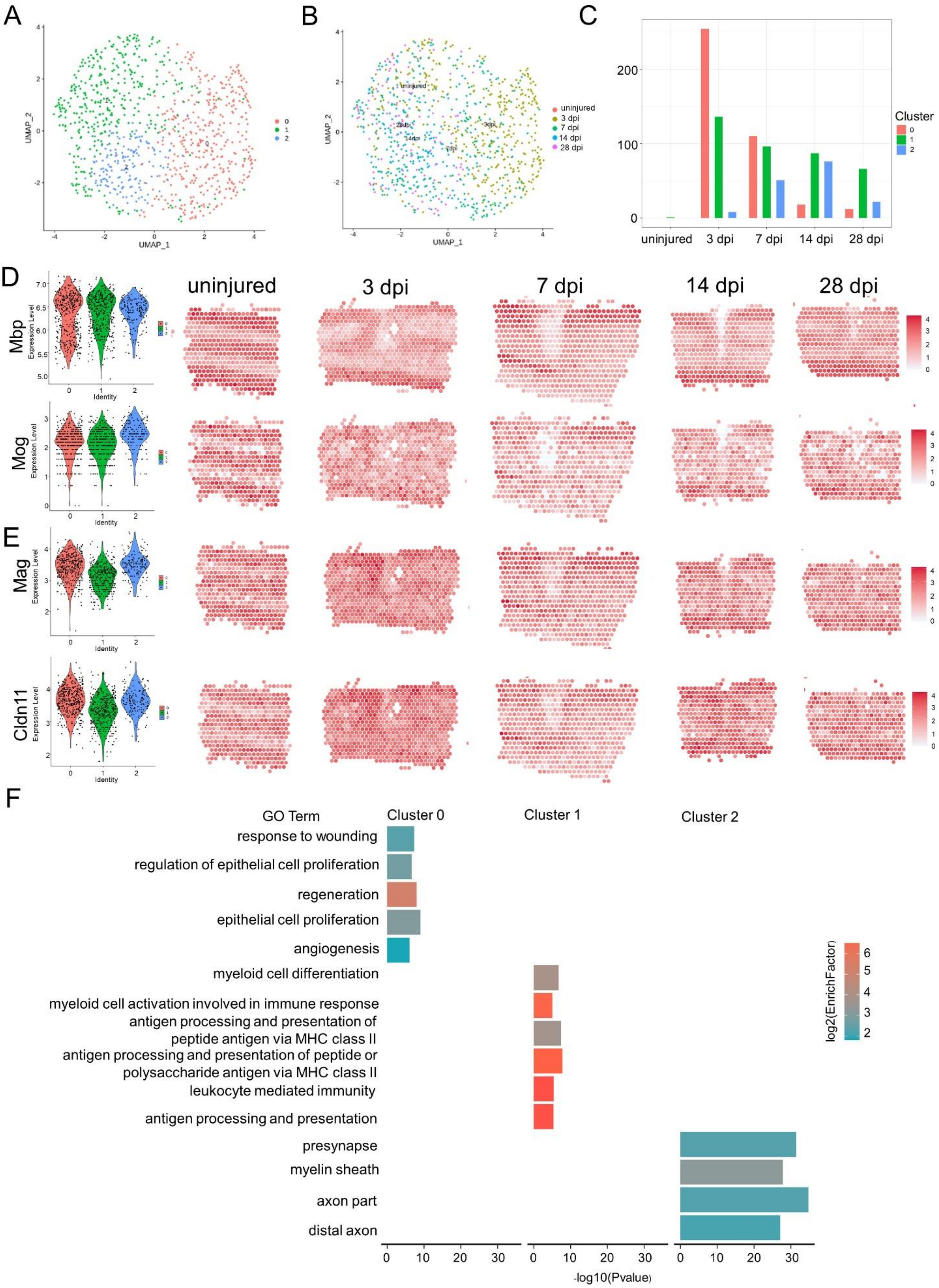

A
B
C
UMAP_1
UMAP_2
uninjured
3 dpi
7 dpi
14 dpi
28 dpi
Cluster
0
1
2
D
E
Mbp
Mog
Mag
Cldn11
Expression Level
Identity
F
GO Term
Cluster 0
Cluster 1
Cluster 2
response to wounding
regulation of epithelial cell proliferation
regeneration
epithelial cell proliferation
angiogenesis
myeloid cell differentiation
myeloid cell activation involved in immune response
antigen processing and presentation of peptide antigen via MHC class II
antigen processing and presentation of peptide or polysaccharide antigen via MHC class II
leukocyte mediated immunity
antigen processing and presentation
presynapse
myelin sheath
axon part
distal axon
log2(EnrichFactor)
-log10(Pvalue)

Fig. S6 Distinct oligodendrocyte responses after lateral hemisection if the spinal cord

A UMAP spots showing the 3 clusters of microglia in the glial scar. **B** UMAP spots embedding overlay showing the distribution of spots at different time points after injury. **C** Histogram showing the number of spots in each subpopulation at different time points after injury. **D, E** Violin plots and spatial maps showing the expression of Mbp and Mog (**D**) and Mag and Cldn11 (**E**) at different time points after injury. **F** Selected GO terms that were enriched for each cluster (Fisher's exact test).

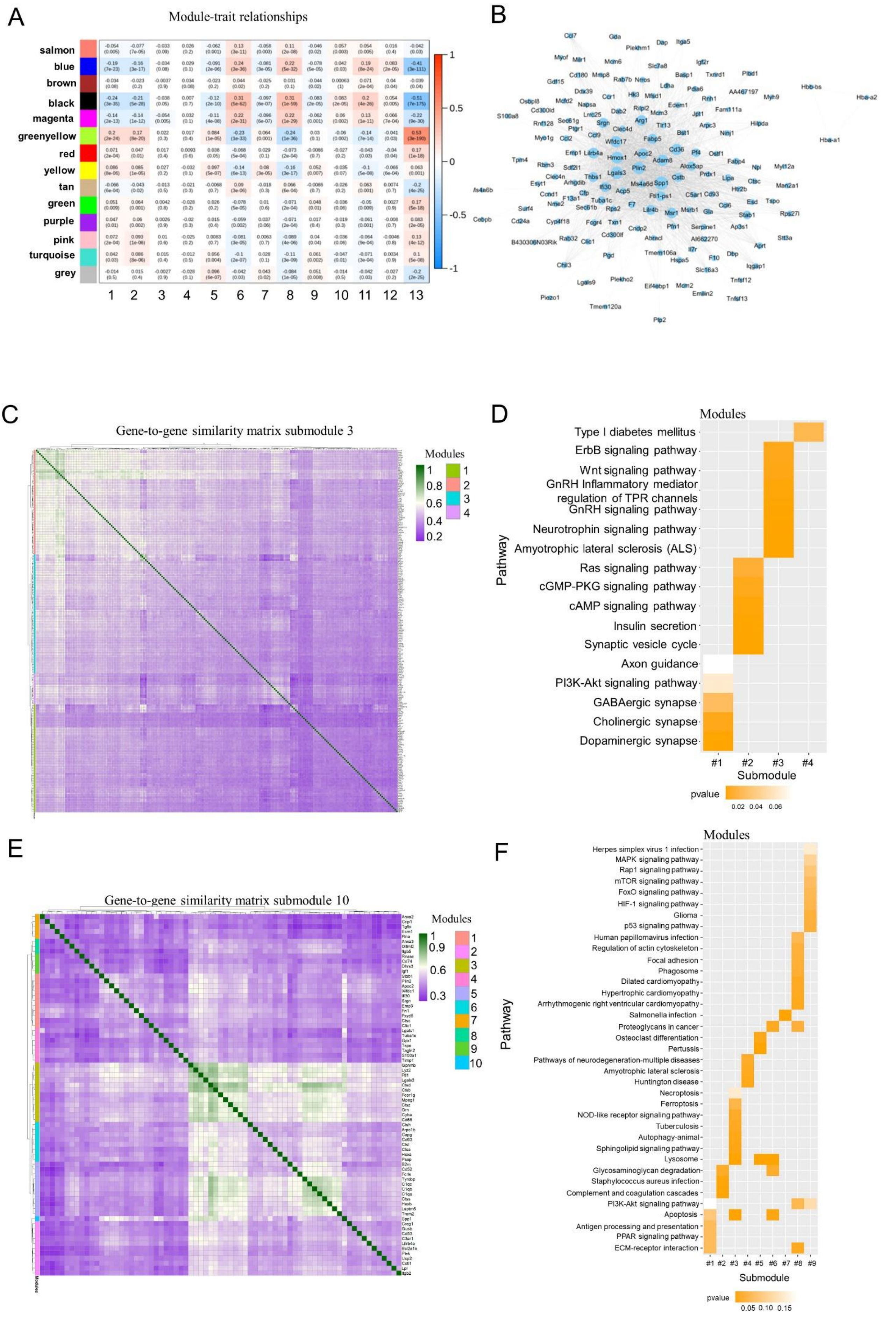
A
Module-trait relationships
salmon
blue
brown
black
magenta
greenyellow
red
yellow
tan
green
purple
pink
turquoise
grey
1 2 3 4 5 6 7 8 9 10 11 12 13
B
C
Gene-to-gene similarity matrix submodule 3
Modules
D
Modules
Type I diabetes mellitus
ErbB signaling pathway
Wnt signaling pathway
GnRH Inflammatory mediator
regulation of TPR channels
GnRH signaling pathway
Neurotrophin signaling pathway
Amyotrophic lateral sclerosis (ALS)
Ras signaling pathway
cGMP-PKG signaling pathway
cAMP signaling pathway
Insulin secretion
Synaptic vesicle cycle
Axon guidance
PI3K-Akt signaling pathway
GABAergic synapse
Cholinergic synapse
Dopaminergic synapse
Pathway
#1 #2 #3 #4
Submodule
pvalue
0.02 0.04 0.06
E
Gene-to-gene similarity matrix submodule 10
Modules
F
Modules
Herpes simplex virus 1 infection
MAPK signaling pathway
Rap1 signaling pathway
mTOR signaling pathway
FoxO signaling pathway
HIF-1 signaling pathway
Glioma
p53 signaling pathway
Human papillomavirus infection
Regulation of actin cytoskeleton
Focal adhesion
Phagosome
Dilated cardiomyopathy
Hypertrophic cardiomyopathy
Arrhythmogenic right ventricular cardiomyopathy
Salmonella infection
Proteoglycans in cancer
Osteoclast differentiation
Pertussis
Pathways of neurodegeneration-multiple diseases
Amyotrophic lateral sclerosis
Huntington disease
Necroptosis
Ferroptosis
NOD-like receptor signaling pathway
Tuberculosis
Autophagy-animal
Sphingolipid signaling pathway
Lysosome
Glycosaminoglycan degradation
Staphylococcus aureus infection
Complement and coagulation cascades
PI3K-Akt signaling pathway
Apoptosis
Antigen processing and presentation
PPAR signaling pathway
ECM-receptor interaction
Pathway
#1 #2 #3 #4 #5 #6 #7 #8 #9
Submodule
pvalue
0.05 0.10 0.15

Fig. S7 Spatiotemporal dynamics of gene expression during maturation of the scar

A Heatmap depicting the correlation scores (digit in the box above) as well as its corresponding P-value (digit in the box below) of modules (rows) and different cell types after injury (columns). 1, astrocytes; 2, microglia; 3, neurons and astrocytes; 4, vascular smooth muscle cells and endothelial cells; 5, fibroblasts; 6, macrophages; 7, astrocytes; 8, oligodendrocytes; 9, astrocytes and oligodendrocytes; 10, endothelial cells; 11, macrophages; 12, fibroblasts; 13, time. **B** Co-expression of selected hub genes enriched in Meblack. The networks were created by Cytoscape. **C** Hierarchical clustering of genes in module 3. **D** Analysis of enriched KEGG pathways among the genes for submodules in **C**. **E** Hierarchical clustering of genes in module 10. **F** Analysis of enriched KEGG pathways among the genes for submodules in **E**.
